# *Helicobacter pylori* VacA modulates TRAF1-mediated 4-1BB/NF-kappaB axis to induce host apoptosis and chronic inflammatory damage

**DOI:** 10.1186/s10020-025-01349-5

**Published:** 2025-10-24

**Authors:** Lingzhi Yuan, Shuoyi Yao, Nanfang Qu, Xinmeng Li, Xueer Yang, Aojian Deng, Minglin Zhang, Ting Cai, Lunxi Liang, Zhitao Liu, Xiaoming Liu, Fen Wang

**Affiliations:** 1 Department of Gastroenterology, the Third Xiangya Hospital, Central South University, Changsha, Hunan 410013 China; 2 Hunan Key Laboratory of Nonresolving Inflammation and Cancer, Central South University, Changsha, Hunan 410013 China; 3 Department of Gastroenterology, Affiliated Hospital of Guilin Medical University, Guilin, Guangxi 541001 China; 4 People‘s Hospital of Ningxiang City, Ningxiang Hospital affiliated to Hunan University of Chinese Medicine, Ningxiang, Hunan 410600 China

**Keywords:** *Helicobacter pylori*, VacA, TRAF1/4-1BB, NF-κB, IL-8, Cell proliferation and apoptosis

## Abstract

**Background:**

*Helicobacter pylori* (Hp) infection is the primary cause of gastric cancer. We previously demonstrated that TRAF1 expression was significantly increased during the multistep pathological timeline of Correa’s cascade and that these changes were closely correlated with Hp virulence factor VacA. However, the underlying mechanism remains unknown.

**Methods:**

TRAF1-deficient or overexpressing human gastric mucosal epithelial cells were generated, and wild-type/*vacA*-KO mutant Hp, recombinant VacA protein and pDsRED2-N1-HA/VacA plasmid were used for mechanistic investigation of VacA. In addition, we established mice infection models and evaluated gastric pathological changes.

**Results:**

RNA sequencing and cellular experiments suggested that positive correlations between TRAF1 expression and NF-κB pathway activation and apoptosis. VacA increased the protein levels of TRAF1, 4-1BB, p-IKKα/β, phosphorylated p65, Bax, and the downstream inflammatory factor IL-8 but reduced that of Bcl-xl, promoting apoptosis and inhibiting proliferation in vitro. Transient and stable overexpression of TRAF1 promoted VacA-induced 4-1BB expression, NF-κB pathways activation, apoptosis-related molecules levels, and downstream inflammatory factors secretion in gastric epithelial cells, whereas silencing of TRAF1 led to the opposite effects. Functionally, TRAF1 increased VacA-induced p65 nuclear translocation and transcriptional activity, leading to apoptosis. BAY11-7082 (an NF-κB inhibitor) or 4-1BB blocking antibody attenuated the promotive effect of TRAF1 overexpression on VacA-induced NF-κB pathway activation, apoptosis-related protein expression, and downstream inflammatory factors secretion in gastric epithelial cells, whereas 4-1BB agonist antibody reversed the inhibitory effect of TRAF1 silencing on VacA function. In vivo, the immunofluorescence of TRAF1, 4-1BB and NF-κB p65 at *vacA*^+^Hp colonization sites was increased, and TRAF1 expression increased gradually with prolonged infection, correlating with exacerbated gastric mucosal inflammation and damage. Hp eradication or BAY11-7082 treatment reversed TRAF1 expression in mouse mucosal tissue.

**Conclusions:**

We revealed a novel VacA–TRAF1–NF-κB pathway–IL-8 signalling axis, exposing novel connections among Hp infection, inflammation and gastric tumorigenesis.

**Graphical Abstract:**

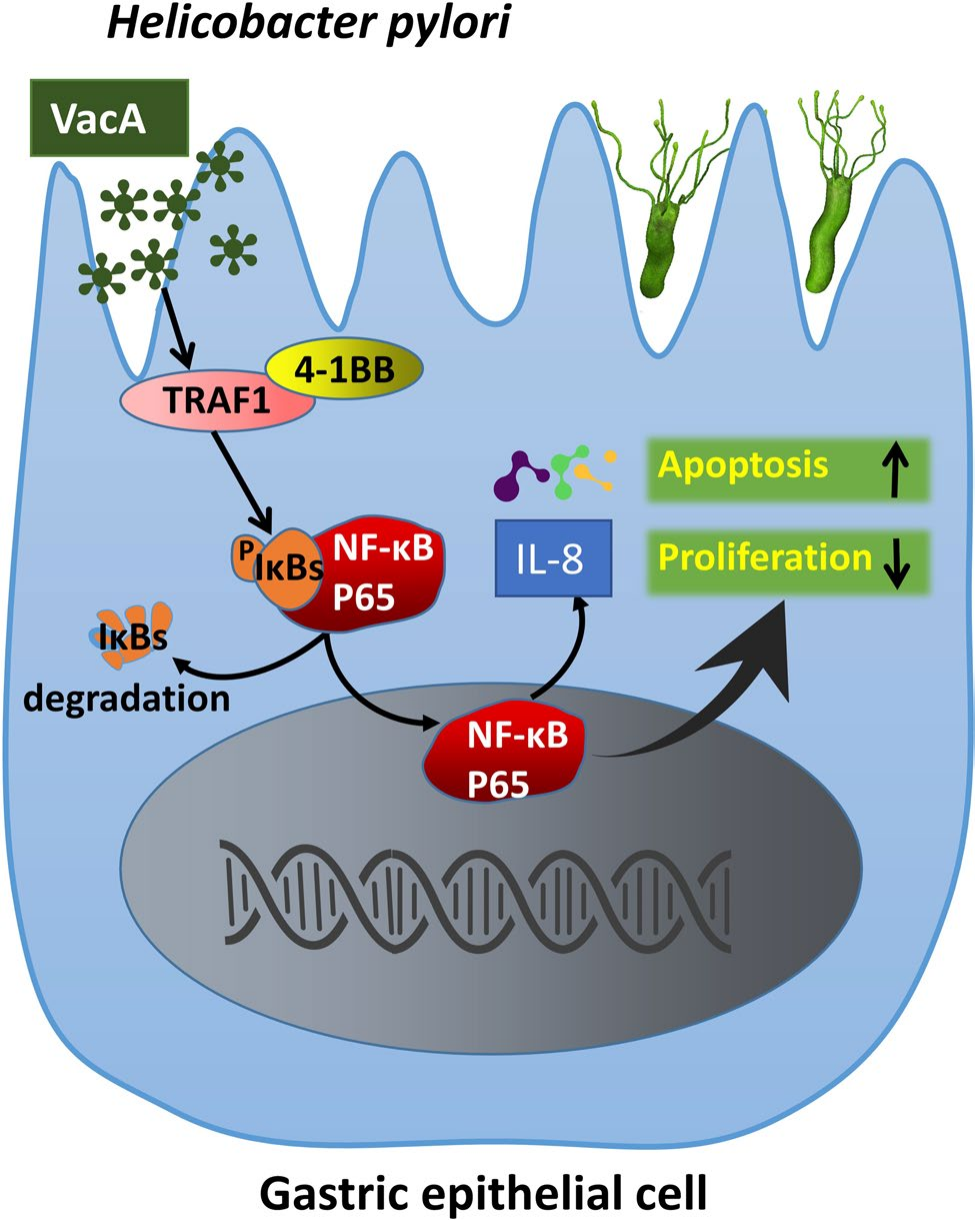

**Supplementary Information:**

The online version contains supplementary material available at 10.1186/s10020-025-01349-5.

## Introduction

*Helicobacter pylori* (Hp) infection, the most widespread chronic bacterial infection in humans, is considered the single most important risk factor for the development of gastric cancer, and Hp is classified as a type I carcinogen by the World Health Organization(WHO)/International Agency for Research on Cancer (Amieva and Peek 2016; Ford et al. 2020). Persistent Hp infection, particularly with highly virulent strains such as *vacA*s1m1, (Ansari and Yamaoka, 2020; Atherton, et al. 1995) results in chronic gastric inflammation, tissue damage, and increased cell proliferation and apoptosis, subsequently leading to precancerous lesions, atrophy, intestinal metaplasia, dysplasia and, ultimately, gastric cancer (Xia and Talley 2001; Cover et al. 2003). Disruption of the balance between cell proliferation and apoptosis by Hp is among the most important hallmarks of Hp infection and triggers the development of Hp-associated gastric diseases (Xia and Talley 2001; Jones et al. 1997; Wang et al. 2014a; Moss et al. 1996). The apoptotic fate could therefore be central to driving tumour development and attenuating immune control. However, to date, the processes by which pro and antiapoptotic host signalling induced by Hp, influencing the complex molecular and cellular interplay within the gastric mucosa and, ultimately, gastric carcinogenesis, are poorly understood.

One of the most crucial toxins produced by Hp is vacuolating cytotoxin A (VacA), which plays a vital role in the pathogenesis of peptic ulcer disease and gastric cancer (Cover and Blanke 2005; Baj et al. 2020). VacA, the only exocrine toxin of Hp, is a multifunctional toxin that acts by causing cell vacuolation and apoptosis. (Ansari, et al. 2020; Baj, et al. 2020; McClain, et al. 2017). Numerous studies have shown that VacA can induce apoptosis through the mitochondrial pathway in gastric epithelial cells (Calore et al. 2010; Jain et al. 2011; Boquet and Ricci 2012). VacA can be transferred to mitochondria, where it causes dissipation of the mitochondrial transmembrane potential, cytochrome c release, and activation of the proapoptotic factor Bcl-2-associated X protein (Bax), thereby leading to apoptosis (Jain et al. 2011; Galmiche et al. 2000). Another study suggested that VacA causes cytochrome c release indirectly by activating the proapoptotic Bcl-2 apoptosis regulator(Bcl-2) family protein Bax (Yamasaki et al. 2006), but the mechanism through which VacA induces Bax activation is not fully understood. Research based on immunostaining and confocal microscopy has shown that the VacA protein is localized predominantly to vacuoles rather than mitochondria (Yamasaki et al. 2006), indicating that VacA may be involved in other pathways leading to cell death.

Tumour necrosis factor receptor-associated factors (TRAFs) were initially discovered as adaptor proteins that couple the tumour necrosis factor receptor family to signalling pathways and are thought to be important regulators of cell death and cellular responses to stress (Bradley and Pober 2001). TRAF1 is an Nuclear factor κB (NF-κB)-inducible protein, and the absence of a RING domain in its N-terminal region, which is present in other TRAFs, makes TRAF1 unique among members of the TRAF family (Edilova et al. 2018). TRAF1 can directly or indirectly interact with multiple Tumor Necrosis Factor Receptor (TNFR) superfamily members, such as 4-1BB[cluster of differentiation (CD) 137/tumour necrosis factor receptor superfamily member 9], regulator proteins, kinases, and adaptors, which contributes to its diverse functions in specific tissues under physiological conditions (Bradley and Pober 2001). TRAF1 plays an important role in the regulation of T-cell activation by limiting NF-κB-inducing kinase (NIK) activation in activated T cells and by promoting 4-1BB-mediated activation of the canonical NF-κB pathway (McPherson et al. 2012). TRAF1 is involved in numerous signalling interactions and plays an important role in many human diseases by regulating apoptosis (Chen et al. 2022; Zhang et al. 2014). However, the functional roles of TRAF1 in these processes have not been fully explained, and studies of its role in the “cellular inflammation-death” lesions caused by Hp infection are especially rare.

In our previous study, GES-1 cells were cocultured with Hp strains isolated from patients with gastric carcinoma or chronic gastritis, and the most virulent strain was identified via in vitro proliferation and apoptosis assays. The most virulent strain was found to be the virulent *vacA*s1m1 Hp strain via Polymerase Chain Reaction (PCR) genotyping (Wang et al. 2011, 2012). Then, using a human whole-genome microarray, we found that coculture of the Hp *vacA*s1m1 strain isolated from patients with gastric cancer with gastric epithelial GES-1 cells resulted in markedly increased expression of TRAF1, 4-1BB, and the chemokine interleukin (IL)−8 (a downstream target of the NF-κB pathway) (Wang et al. 2011, 2012, 2015). Further clinical studies demonstrated that TRAF1 and 4-1BB expression is markedly upregulated in tissues of intestinal metaplasia with atypical hyperplasia and in gastric cancer tissues and that these effects are associated with Hp *vacA*s1m1 infection (Wang et al. 2014b, 2015). These previously published data indicate that the upregulation of TRAF1 and 4-1BB expression is associated with Hp *vacA*s1m1 infection and contributes to the increased carcinogenicity of Hp *vacA*s1m1, but the underlying mechanism remains unclear. In this work, we aimed to reveal the existence of the VacA–TRAF1–NF-κB pathway–IL-8 axis in gastric epithelial cells and the mechanism of gastric carcinogenesis induced by the Hp virulence factor VacA.

## Materials and methods

### Cell lines and treatments

Three human gastric mucosal epithelial cell lines (GES-1, HGC27, MKN74) were were obtained from cell bank of Chinese Academy of Sciences (Shanghai, China), and cultured in RPMI-1640 mediu mcontaining 10% fetal bovine serum. All the cells were identifed by short tandem repeat (STR). NF-κB inhibitor BAY11-7082 (BAY) (Selleck, USA), Anti-human 4-1BB Blocking Antibody (BioLegend, USA) and Anti- 4-1BB Agonist Antibody (BPS Bioscience, USA) were used at the dose as indicated, cells were pretreated for 2 h.

### Establishment of stable TRAF1 knockdown and overexpression cell lines

To construct stable TRAF1-deficient gastric mucosal epithelial cells, MKN74 cells were infected with shTRAF1 at a multiplicity of infection (MOI) of 10. After 48 h of infection, the cells were incubated with puromycin (2 µg/mL) for 14 days to select the stable expression cells, and the stable TRAF1-knockdown clones were validated by Western blot analysis and Quantitative real-time polymerase chain reaction (qRT–PCR), whereas the cells stably transfected with scramble control Short Hairpin RNA (shRNA) vector were used as the negative control. Meanwhile, stable TRAF1-overexpressing HGC27 cells were also constructed using the lentiviral system.

### Transient transfection

GES-1 cells were used to construct transient transfected gastric mucosal epithelial cells. According to the instructions of Lipofectamine 3000 (Life Technologies, Rockville, MD, USA), VacA-overexpressing plasmids (full-length cDNA encoding Hp J99, pDsRED2-N1-HA as carrier) and no-load control plasmids were transfected into GES-1 cells. After 6 h of culture in serum-free medium, the culture was transferred to 10% serum medium for 48 h. Cells were collected for subsequent experiments. Plasmids (expression plasmid pcDNA3.1-TRAF1, empty vector pcDNA3.1) or siRNAs against Transgelin and corresponding negative control siRNAs were transfected with the same method. The efficiency of transfection was evaluated by western blot.

### Hp strains and infection of gastric cells

The wild-type strain Hp American Type Culture Collection (ATCC) 26695 (*vacA*+) and *vacA*-KO mutant Hp ATCC 26695 (Δ*vacA*; Quanming Zou and Chunhui Lan, Department of Gastroenterology, Daping Hospital, Third Military Medical University) were cultured on Campylobacter agar plates containing 10% sheep serum at 37 °C under microaerophilic conditions (5% O_2_, 10% CO_2_, and 85% N_2_). For cell infection assays, Hp was washed from the culture plates with sterile phosphate‐buffered saline, centrifuged at 2500 × g for 5 min, and resuspended in RPMI‐1640 medium. Once human gastric epithelial cells reached 70% confluence, they were cocultured with Hp strains for different times or at different multiplicities of infection (MOI; a bacteria‐to‐cell ratio). The relative bacterial number was determined by measuring the optical density at 600 nm (1 OD600 = 1 × 10^9^ CFU/mL). (CFU, Colony Forming Unit)

### Mice and Hp strains infection model

All animal care and experimental protocols were in accordance with guidelines established by the Institutional Animal Care and Use Committee Center of Central South University (approval ID: CSU-2022-0001-0257). C57BL/6 mice (female, six weeks old, 16–22 g) purchased from the Laboratory Animal Science Center of Central South University and maintained in an isolated clean room with aregulated temperature (20–22 °C), humidity (approximately 55%), and 12/12-h light/dark cycle with ad libitum rodent diet and water. After one week of observation, the C57BL/6 mice were fasted for 12 h prior to the challenge. Subsequently, the mice were challenged using orogastric infusions of 300 µl of sterile Brucella broth, 5 × 10^9^ CFU/ml *vacA*^+^ Hp strain (the wild-type strain Hp ATCC 26695) or 5 × 10^9^ CFU/ml*ΔvacA* Hp strain (*vacA*-KO mutant Hp ATCC 26695) once every 2 days for a total of 9 infusions. Two weeks after the final gastric lavage with Hp in C57BL/6 mice, we randomly selected three mice from each group to confirm the colonization of Hp in the gastric mucosa through Giemsa staining, immunohistochemical staining, and the rapid urease test. C57BL/6 mice underwent triple anti-Hp therapy six months after infection with either the *vacA*^*+*^Hp or*ΔvacA* Hp strain. The triple therapy consisted of omeprazole (40 µmol/kg/d), clarithromycin (7.15 mg/kg/d), and metronidazole (14.2 mg/kg/d) administered for 14 consecutive days. After 1 and 3 months of Hp eradication treatment, the efficacy of the anti-Hp therapy was verified through urease testing and Giemsa staining. The animals were euthanized at 1,3,6 months post-challenge, and the gastric tissues were harvested for pathology and western blots analyses, blood samples were collected from the tail vein for Enzyme-Linked Immunosorbent Assay (ELISA) analysis.

### Recombinant VacA protein

The expression and purification of recombinant VacA protein were performed as previously described (Karin and Lin 2002). Briefly, the *vacA* gene of Hp J99 (ATCC 700824, *cagAvacA*s1m1) was amplified from the sequence (a mature protein fragment of the VacA toxin consisting of amino acids 34 to 858; the first 33 amino acids of the *vacA* gene product resemble bacterial signal peptides and were deleted) by PCR. *E. coli* strain TOP10 (Invitrogen) was used for subcloning. The PCR product was digested with NdeI and XhoI and inserted into the expression vector pET41b containing a C-terminal histidine tag (8His.tag; Novagen Company). Then, the identified recombinant VacA plasmid was transformed into competent *E. coli* BL21(Escherichia coli λ lysogen DE3) cells (Novagen Company). The recombinant VacA protein was then purified from the inclusion bodies by Nickel(II) Nitrilotriacetate (Profinity IMAC Ni-Charged Resin, Bio-Rad Laboratories, Inc.) and analysed by Sodium Dodecyl Sulfate-Polyacrylamide Gel Electrophoresis (SDS–PAGE) and Western blotting. Finally, the 89.7-kDa recombinant VacA^34–854^ protein (purity > 90%) was refolded and stably preserved in 50 mM acetic acid buffer (pH 2.9) (Yuan et al. 2021). 

### Recombinant VacA protein incubation cell model

Gastric mucosal cells were seeded into 6-well plates at a density of 5 × 10^6^ cells/well. After reaching 70% confluence, the cells were incubated with different concentrations of recombinant VacA protein for a specified time in a humidified atmosphere with 5% CO2 at 37 °C. Simultaneously, other batches of gastric mucosal cells were incubated with RPMI-1640 medium or isovolumetric protein buffer (acetic acid) to serve as an untreated normal group and a buffer control group, respectively.

### Treatment of mice with Recombinant VacA protein

The C57BL/6 mice were fasted for 12 h and dehydration for 4 h prior to the challenge. Each mouse was pretreated with 0.06 ml of 50% ethanol by gavage. C57BL/6 mice were randomly divided into four groups (blank control group, VacA recombinant protein gavage group, acetic acid buffer gavage group, Bovine Serum Albumin (BSA) standard protein gavage group, and VacA recombinant protein gavage group). VacA recombinant protein was gavaged at a dose of 10 mg/kg, while the control group was gavaged with an equal amount of acetic acid buffer or BSA standard protein once a day for 14 consecutive days. The four groups of mice were given corresponding BAY11-7082 treatment groups, that is, C57BL/6 mice were pre treated with BAY11-7082 intraperitoneal injection (5ug/kg) once a day for 14 consecutive days.

### RNA sequencing

Total RNA was isolated using RNAiso Plus (Total RNA extraction reagent, Thermo Fisher Scientific) according to the manufacturer’s instructions and quantified by the NanoDrop 2000 spectrophotometer. TruSeq Stranded Total Sample Preparation Kit (Illumina, 20020596) was used for sequencing library construction. Libraries were deeply sequenced on the Illumina Novaseq 6000 according to the activity and expected data volume. The raw RNA Sequencing (RNA-Seq) sequence reads were trimmed using Trimmomatic (version 0.39) to remove low-quality reads and adapters. Trimmed data were first evaluated by the software “FastQC” (https://www.bioinformatics.babraham.ac.uk/projects/fastqc/) with the default parameter and then aligned with Hisat2 (version 2.1.0) against the human (hg38) genome guided by Genome Reference Consortium gene annotation (version 34) with the default parameter. The abundance of genes in each sample was calculated by StringTie packages (version 2.1.2) with the “-e” parameter. Differentially expressed genes were identified using the R package DESeq2 (version 1.34.0) with the following condition: adjusted p-value < 0.05 and the absolute value of log2 foldchange > 1. The raw data of the RNA-seq was deposited in the Gene Expression Omnibus (GEO) database (GSE290844).

### Cell viability assays

Cell viability was determined using an 3-(4,5-Dimethylthiazol-2-yl)−2,5-diphenyltetrazolium Bromide (MTT) assay or a Cell Counting Kit-8 (CCK-8) assay. The cells were seeded at a density of 2 × 10^3^ cells/well in 96-well plates. Twelve hours after seeding, the cells were transfected with plasmid or incubated with recombinant VacA protein. At the indicated time points, 20 µl of MTT solution or 100 µl of CCK-8 solution was added to each well, and the cells were cultured for an additional 4 h. Cell viability was quantified by measuring the absorbance at 490 or 450 nm using a microplate spectrophotometer to calculate optical density (OD) values.

### Cellular apoptosis assay

Cellular apoptosis was assayed by flow cytometry using a Fluorescein Isothiocyanate (FITC) Annexin V Apoptosis Detection Kit I (cat. no. 556547, BD Biosciences) according to the manufacturer’s instructions. Following incubation or transfection, the cells were harvested and resuspended in cold Phosphate Buffered Saline (PBS). After centrifugation at 94 × g and 4 °C for 5 min, the cells were resuspended in 500 µl of binding buffer and mixed with 5 µl of Annexin V-FITC. The cells were subsequently incubated with 5 µl of propidium iodide (PI) in the dark at room temperature for 15 min. The samples were analysed with a FACSCanto II flow cytometer (BD Biosciences) and FlowJo software.

### Quantitative real-time polymerase chain reaction (qRT–PCR)

Total RNA (2 µg) was extracted using TRIzol reagent (Thermo Fisher Scientific, Inc., Waltham, MA, USA) according to the manufacturer’s protocol and reverse transcribed in a 20-µl reaction system using a RevertAid First Strand cDNA Synthesis Kit (Thermo Fisher Scientific, IncWaltham, MA, USA). qPCR was performed using SYBR Premix ExTaq™ (Takara Biotechnology Co., Ltd., Dalian, China) reagents according to the manufacturer’s protocol. The following primer sequences were used: VacA forward, 5’-ATGCGGGTTATGCCAGACAA-3’, and VacA reverse, 5’-TAGCGTTGAGCGAACGAGTTA-3’; TRAF1 forward, 5’-TCCCGTAACACCTGATTAA-3’, and TRAF1 reverse, 5’-ACAACTCCCAAACCATACAC-3’; 4-1BB forward, 5’-CGTGGTCTGTGGACCATCTC-3’, and 4-1BB reverse, 5’-ACAACAGAGAAACGGAGCGT-3’; IL-8 forward, 5’-CCAGGAAGAAACCACCGGAA-3’, and IL-8 reverse, 5’-TTCCTTGGGGTCCAGACAGA-3’; and GAPDH forward, 5’-AACGGATTTGGTCGTATTGGG-3’, and GAPDH reverse, 5’-TCGCTCCTGGAAGATGGTGAT-3’. The conditions were as follows: predenaturation at 95 °C for 3 min and 40 cycles of 95 °C for 10 s and 60 °C for 30 s. The relative expression levels of VacA, TRAF1, 4-1BB and IL-8 were normalized to the GAPDH level and calculated using the 2-∆∆Cq method.

### Western blot analysis

Total protein was extracted from cells using lysis buffer containing 20 mM Tris-HCl (pH 7.4), 150 mM NaCl, 5 mM Ethylene Diamine Tetraacetic Acid (EDTA), 1% Triton-X 100, 1% Dithiothreitol and 1% protease inhibitor cocktail (Roche Diagnostics, Basel, Switzerland). Nuclear and cytoplasmic proteins were extracted using a nuclear and cytoplasmic protein extraction kit (Beyotime Institute of Biotechnology, Shanghai, China) according to the manufacturer’s protocol. The protein concentration was measured using a BCA protein assay kit (Thermo Fisher Scientific, Inc.). Equal amounts of protein extracts (50 µg) were separated by SDS–PAGE on a 10% gel, and the proteins were transferred onto a polyvinylidene fluoride membrane. The membranes were blocked with 5% w/v non-fat dried milk dissolved in TBS with Tween-20 (TBS-T; 0.1% Tween-20, pH 8.3) at room temperature for 1 h and incubated with primary antibodies overnight at 4 °C. The following primary antibodies were used: anti-VacA (1:500, sc-32746, Santa Cruz Biotechnology), anti-p-IKKα/β (1:500, 2697, Cell Signaling Technology, CST), anti-p-P65 (1:500, 3033S, CST), anti-P65 (1:1000, 4764, CST), anti-TRAF1 (1:500, 4715, CST), anti-4-1BB (1:100, ab203391, Abcam), anti-Caspase-9 (1:500,9508,CST), anti-Caspase-3 (1:500, 9662, CST), anti-Bcl-xl (1:1000, 2762,CST), anti-Bax (1:1000, 2774, CST), anti-histone-H3 (1:1000, 17168–1-AP, Proteintech) and anti-Glyceraldehyde-3-phosphate dehydrogenase(GAPDH) (1:5000, MAB374, Merck KGaA). The membranes were washed with TBS-T and incubated with HRP-labelled anti-rabbit or mouse IgG secondary antibody (both 1:5,000, sc-2030 or sc-2302, CST) for 1 h at room temperature. Bands were visualized using an enhanced chemiluminescence kit (EMD Millipore) and the ChemiDoc XRS system (Image Lab™ software version 4.0; Bio-Rad Laboratories, Inc., Hercules, CA, USA).

### ELISA

Following infection, incubation or transfection, the cell culture medium was collected and the amount of IL-8, IL-6, and TNFα in the cell culture supernatant were detected using the corresponding Enzyme-Linked Immunosorbent Assay (ELISA) kits, respectively (CHE0011, CHE0009, CHE0019, Beijing 4A Biotech Co., Ltd) according to the manufacturer’s protocol. Similarly, the levels of IL-8 in mouse blood samples were measured with the IL-8 ELISA kit (CME0008, Beijing 4A Biotech Co., Ltd). The OD of each well was read using a microplate reader at a wavelength of 450 nm.

### Immunofluorescence staining

The cells or gastric mucosa tissue were fixed with 4% paraformaldehyde, then the cells or gastric mucosa tissue slides were permeabilized with 0.5% Triton X-100, and then blocked with 5% BSA. The primary antibodies were incubated overnight at 4℃, followed by incubation with appropriate goat anti-rabbit secondary antibodies or fuorescent dye-labeled secondary antibodies at room temperature for 2 h. The nuclei were stained with 4, 6-diamidino-2-phenylindole (DAPI) (Sigma, USA), and the stained cells were imaged with a Leica TCS Inverted Fluorescence Microscope. For statistical analysis, the number of positive cells was counted at the corresponding magnification in 5 random felds. The following primary antibodies were used: anti-Hp (1:100, ab-20459, Abcam), anti-P65 (1:100, 4764, CST), anti-TRAF1 (1:100, 4715, CST), anti-4-1BB (1:50, ab203391, Abcam), anti-Bcl-xl (1:100, 2762, CST).

### Immunohistochemistry staining

Tissue specimens were fxed in 10% formalin solution and embedded in parafn wax, then 5 μm serial sections were cut from the tissue blocks, deparafnized in xylene, and dehydrated in a series of alcohol concentrations (75%, 85%, 95%, 100%), followed by antigen retrieval with ethylene EDTA or citrate bufer and blocked with 5% goat serum. Tissue sections were then incubated with primary antibodies against Hp (1:100, ab-20459, Abcam), P65 (1:200, 4764, CST), TRAF1 (1:100, 4715, CST), 4-1BB (1:50, ab203391, Abcam), Bcl-xl (1:100, 2762, CST), Bax (1:200, 2774, CST). Subsequently, tissue sections were incubated with secondary antibodies (peroxidase-conjugated goat anti-rabbit IgG, A-11008, Invitrogen) for 2 h at room temperature, and stained with 3,3’-Diaminobenzidine (DAB) kit (Sigma, USA). After staining, sections were digitally scanned using the Aperio AT2 scanner (Leica Biosystems, Germany), and analyzed with Aperio image analysis workstation using a pathologist-trained nuclear, membranal, and nuclear & cytoplasmic algorithms. Protein expression was evaluated according to the H-Score obtained from Aperio image analysis workstation.

### Statistical analysis

All data were analysed using Statistical Package for the Social Sciences (SPSS) software (version 21.0; SPSS, Inc., Chicago, IL, USA). The data are expressed as the means ± standard error of mean The standard two-tailed Student’s t-test was used for statistical analysis, in which *p* < 0.05 was considered statistical significance.

## Results

### TRAF1 promotes the expression of 4-1BB, p-IKKα/β, p-p65, apoptosis-related proteins and downstream chemokines in gastric epithelial cells

**Fig. 1:**
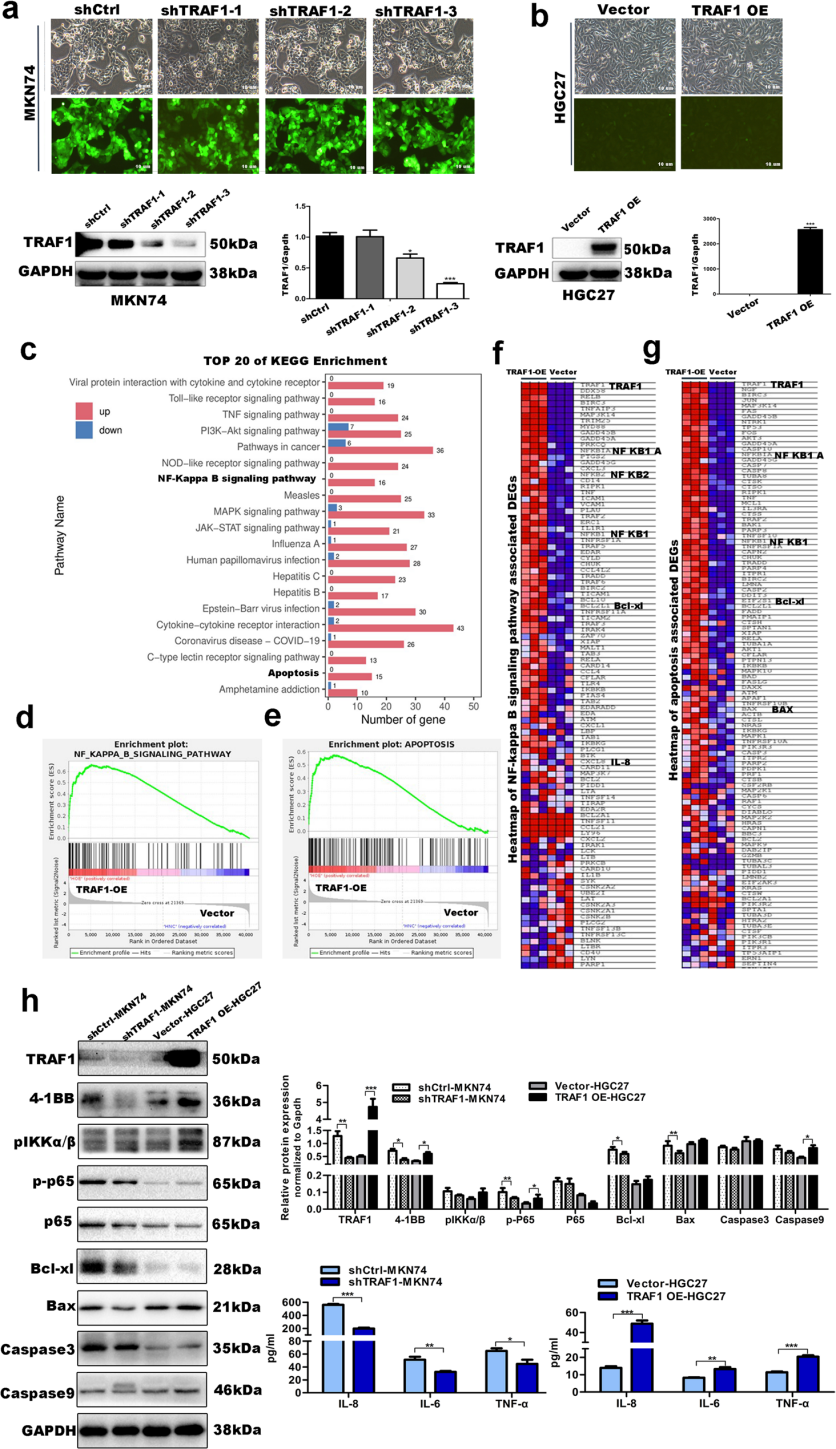
TRAF1 promotes the expression of 4-1BB, molecules of NF-κB pathway, apoptosis-related proteins, and downstream inflammatory factors in gastric mucosal epithelial cells. **a** Construction of stable TRAF1-silenced cell lines. The fluorescence intensity of each cell line was observed by fluorescence microscopy after infection of MKN74 cells with interfering lentivirus and control lentivirus (100X). The silencing effect of TRAF1 in MKN74 stable cell lines was detected by Western blot and qRT-PCR. shTRAF1-1, shTRAF1-2, and shTRAF1-3 represent cells infected with lentivirus corresponding to three different TRAF1 interference sequences, respectively; shCtrl represents cells infected with control virus. GAPDH was used as an internal reference. The relative expression level of TRAF1 (TRAF1/GAPDH): shTRAF1-2 vs. shCtrl, *p* = 0.022; shTRAF1-3 vs. shCtrl, *p* < 0.001. **b** Construction of stable TRAF1-overexpressing cell lines. The fluorescence intensity of each cell line was observed by fluorescence microscopy after infection of HGC27 cells with overexpressing lentivirus and control lentivirus (100X). The overexpression effect of TRAF1 in HGC27 stable cell lines was detected by Western blot and qRT-PCR. The relative expression level of TRAF1 (TRAF1/GAPDH): TRAF1 OE vs. vector, *p* < 0.001. **c-g** Transcriptome sequencing analysis of TRAF1 stably overexpressing gastric mucosal epithelial cells and their negative control cells. **c** Top 20 differential factors obtained by KEGG enrichment analysis. **d** NF-κB pathway-related GSEA enrichment analysis. **e** Apoptosis-related GSEA enrichment analysis. **f** Heatmap of differential genes related to the NF-kappa B signaling pathway. **g** Heatmap of differential genes related to apoptosis. **h** Western Blot analysis of the effects of stable silencing and overexpression of TRAF1 on 4-1BB, NF-κB pathway, and apoptosis-related proteins in gastric mucosal epithelial cells. GAPDH was used as an internal reference. ELISA detection of inflammatory factors IL-8, IL-6, and TNF-α in the supernatant of TRAF1 stably overexpressing/silenced gastric mucosal epithelial cell lines. The relative protein expression level of TRAF1, 4-1BB, p-p65, Bcl-xl and Bax were compared between shTRAF1-MKN74 cells and shCtrl-MKN74 cells, with statistically significant differences (*p* = 0.0039, *p* = 0.033, *p* = 0.007, *p* = 0.048, *p* = 0.005, respectively). The relative protein expression level of TRAF1, 4-1BB, p-p65, and Caspase9 were compared between TRAF1 OE-HGC27 cells and vector-HGC27 cells, with statistically significant differences (*p* < 0.001, *p* = 0.041, *p* = 0.046, *p* = 0.031, *p* = 0.042, respectively). The level of IL-8, IL-6, and TNF-α were compared between shTRAF1-MKN74 cells and shCtrl-MKN74 cells, with statistically significant differences (*p* < 0.001, *p* = 0.007, *p* = 0.042, respectively). The level of IL-8, IL-6, and TNF-α were compared between TRAF1 OE-HGC27 cells and vector-HGC27 cells, with statistically significant differences (*p* < 0.001, *p* = 0.007, *p* < 0.001, respectively). Note: Comparisons were made between two groups using standard two-tailed Student’s t-test. The figure presents the average of three independent experiments (*n* = 3). Data are presented as mean ± SEM. Error bars represent standard error of mean.*: *p* < 0.05.**; *p* < 0.01.***; *p* < 0.001. The phosphorylation site of Phospho-NF-κB p65 is serine 536. shCtrl, short hairpin control; shTRAF1, short hairpin TRAF1; OE, overexpression

To explore the role of TRAF1 in gastric diseases progression, stable silenced or overexpressing human gastric epithelial cells were generated with lentiviral vectors. We selected gastric epithelial cells with high background expression of TRAF1, namely MKN74, and transfected with three independent short hairpin TRAF1 (shTRAF1). The Short Hairpin RNA (shRNA) with the highest silencing efficiency, namely shTRAF1-3, was selected for further study (Fig. 1a). Meanwhile, gastric epithelial cells with low background expression of TRAF1, namely HGC27, were selected to construct stable overexpression (OE) cell lines of TRAF1 (Fig. 1b).

We conducted transcriptome sequencing on HGC27 cells with stable TRAF1 overexpression and their negative control counterparts. Our Kyoto Encyclopedia of Genes and Genomes (KEGG) analysis revealed that among the top 20 differential pathways, the NF-κB pathway and apoptosis were prominently enriched (Fig. 1c). Gene Set Enrichment Analysis (GSEA) enrichment analysis further corroborated that both the NF-κB pathway and apoptosis exhibited significant enrichment upon TRAF1 overexpression, indicating a positive correlation between TRAF1, the NF-κB pathway, and apoptosis (Fig. 1d-e). A closer examination of the heatmap depicting differentially expressed genes within the NF-κB pathway and apoptosis highlighted proteins such as Bcl-xl and Bax, further affirming the intimate relationship between TRAF1, NF-κB pathway activation, and gastric mucosal epithelial cell apoptosis (Fig. 1f-g). Subsequently, we examined the impact of stable TRAF1 interference in gastric mucosal epithelial cell lines on 4-1BB, NF-κB pathway activation, apoptosis-related genes, and downstream inflammatory factors through Western Blot and ELISA analyses (Fig. 1h). Our findings indicated that silencing TRAF1 in MKN74 cells led to downregulation of 4-1BB expression, decreased expression of NF-κB pathway molecules (p-IKKα/β, p-p65), reduced levels of apoptosis-related proteins (Caspase9, Caspase3, and BAX), and diminished secretion of downstream chemokines (IL-8, IL-6, and TNF-α). Conversely, TRAF1 overexpression in HGC27 cells upregulated 4-1BB expression, enhanced the expression of NF-κB pathway molecules, increased apoptosis-related proteins, and elevated the secretion of the aforementioned chemokines. These results strongly suggest that TRAF1likely play an important role in gastric mucosal epithelial cell apoptosis via the NF-κB pathway.

### VacA alters the expression of TRAF1 and apoptosis-related proteins and upregulates 4-1BB, phospho-p65 and IL-8 expression in gastric epithelial cells

**Fig. 2:**
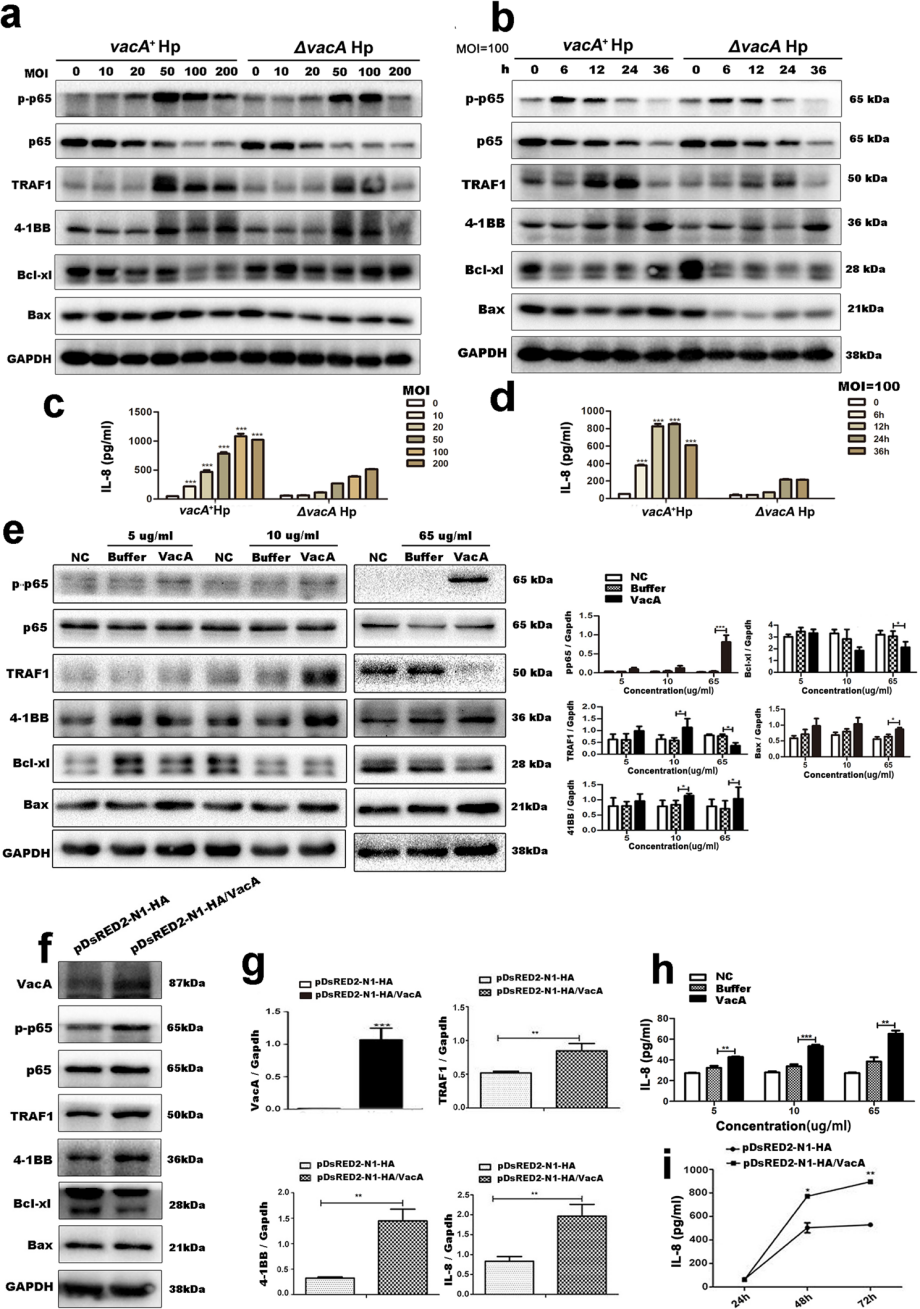
Effects of VacA on TRAF1/4-1BB/NF-κB pathway/chemokine IL-8 axis- and apoptosis-related protein expression in GES-1 cells. **a-d** GES-1 cells infected with *vacA*^*+*^Hp strain and Δ*vacA* Hp strain at different MOIs (0, 10, 20, 50, 100, and 200) for 24 h or different time points (0 h, 6 h, 12 h, 24 h, and 36 h), MOI = 100. **a, b **Western blot analysis of the target proteins (phospho-p65, p65, TRAF1, 4-1BB, apoptosis-related protein Bcl-xl and Bax) levels. GAPDH was used as an internal reference. **c, d** the secretion levels of IL-8 from GES-1 cells after infection were measured by ELISA. Comparison between *vacA*^*+*^Hp strain-infected group and Δ*vacA* Hp strain-infected group under identical MOI conditions (c) or identical intervention time (d) showed statistical significance (*p* < 0.001 for all comparisons). **e** Western blot analysis of the target proteins levels in GES-1 cells incubated with recombinant VacA protein at low concentrations (5 µg/ml and 10 µg/ml) for 48 h or at high concentrations (65 µg/ml) for 12 h. In addition, GES-1 cells incubated with isovolumetric protein buffer and cell culture medium served as a buffer control group and an untreated normal group, respectively. The relative protein expression level of TRAF1 and 4-1BB were compared between recombinant VacA protein-incubated group (10 µg/mL) and the correspondind buffer-incubated group, with statistically significant differences (*p* = 0.041, *p* = 0.037, respectively). The relative protein expression level of p-p65, TRAF1, 4-1BB, Bcl-xl and Bax were compared between recombinant VacA protein-incubated group (65 µg/mL) and the correspondind buffer-incubated group, with statistically significant differences (*p* < 0.001, *p* = 0.039, *p* = 0.044, *p* = 0.049, *p* = 0.047, respectively). **f** Western blot analysis of VacA, phospho-p65, p65, TRAF1, 4-1BB and apoptosis-related protein (Bcl-xl, Bax) levels in GES-1 cells transfected with pDsRED2-N1-HA/VacA for 48 h. **g** qRT‒PCR analysis of VacA, TRAF1, 4-1BB and IL-8 levels in GES-1 cells transfected with pDsRED2-N1-HA/VacA for 48 h. GAPDH was used as an internal control. Comparison of relative expression levels of VacA, TRAF1, 4-1BB, and IL-8 between the pDsRED2-N1-HA-VacA-transfected groups and the empty vector pDsRED2-N1-HA-transfected groups showed statistically significant differences (*p* < 0.001, *p* = 0.007, *p* = 0.009, *p* = 0.005, respectively). **h, i** The secretion level of IL-8 in GES-1 cells after incubation with recombinant VacA protein (**h**) or transfection with pDsRED2-N1-HA/VacA (**i**) was measured by ELISA. **h** Levels of IL-8 were compared between recombinant VacA protein-incubated groups (at concentrations of 5, 10, and 65 µg/mL) and the correspondind buffer-incubated groups, with statistically significant differences (*p* = 0.007, *p* < 0.001, *p* = 0.005, respectively). **i** Levels of IL-8 were compared between the pDsRED2-N1-HA-VacA-transfected groups (for 48 h and 72 h) and the empty vector pDsRED2-N1-HA-transfected groups, with statistically significant differences (*p* = 0.039 and *p* = 0.0071). The figure presents the average of three independent experiments (*n* = 3). Note: Comparisons were made between two groups using standard two-tailed Student’s t-test. The figure presents the average of three independent experiments (*n* = 3). Data are presented as mean ± SEM. Error bars represent standard error of mean. *: *p* < 0.05. **: *p* < 0.01. ***: *p* < 0.001

To further explore the mechanism of the Hp virulence factor VacA, we infected GES-1 cells with the *vacA*^*+*^Hp strain or the Δ*vacA* Hp strain at different multiplicities of infection (MOIs) for 24 h or at different time points (MOI = 100). The Western blot data revealed that the protein levels of TRAF1, 4-1BB, phospho-p65 and proapoptotic Bax protein were significantly increased, whereas that of antiapoptotic Bcl-xl protein was decreased in GES-1 cells infected with the *vacA*^*+*^Hp strain compared with those in cells infected with the Δ*vacA* Hp strain (Fig. 2a-b). Notably, the protein expression of TRAF1 first increased but then decreased with increasing bacterial concentration (Fig. 2a) or increasing infection time (Fig. 2b). Moreover, the ELISA data revealed that the release of IL-8 from GES-1 cells increased in an MOI-dependent or time-dependent manner after *vacA*^*+*^Hp infection (Fig. 2c-d).

Similarly, after incubation with recombinant VacA protein (at low concentrations, 5 µg/ml and 10 µg/ml) or transfection with the pDsRED2-N1-HA-VacA plasmid for 48 h, the protein expression levels of TRAF1, 4-1BB, phospho-p65, and Bax upregulated (Fig. 2e-h), and the IL-8 level in the supernatant increased (Fig. 2i-j). These results are consistent with the previously described effects of bacterial infection. Notably, the expression of TRAF1 decreased after stimulation with the recombinant VacA protein at a higher concentration (65 µg/ml, Fig. 2e-f), and this finding was also consistent with the results of the bacterial infection model described above. Collectively, these results indicate that VacA alters the expression of TRAF1 and increases the levels of 4-1BB, phospho-p65, and the proapoptotic proteins Bax and IL-8 while decreasing the level of the antiapoptotic protein Bcl-xl in gastric epithelial cells. Therefore, VacA is likely to induce abnormal cell proliferation and apoptosis by altering the expression of TRAF1 in gastric epithelial cells.

### VacA activates the NF-κB signalling pathway in gastric epithelial cells

**Fig. 3:**
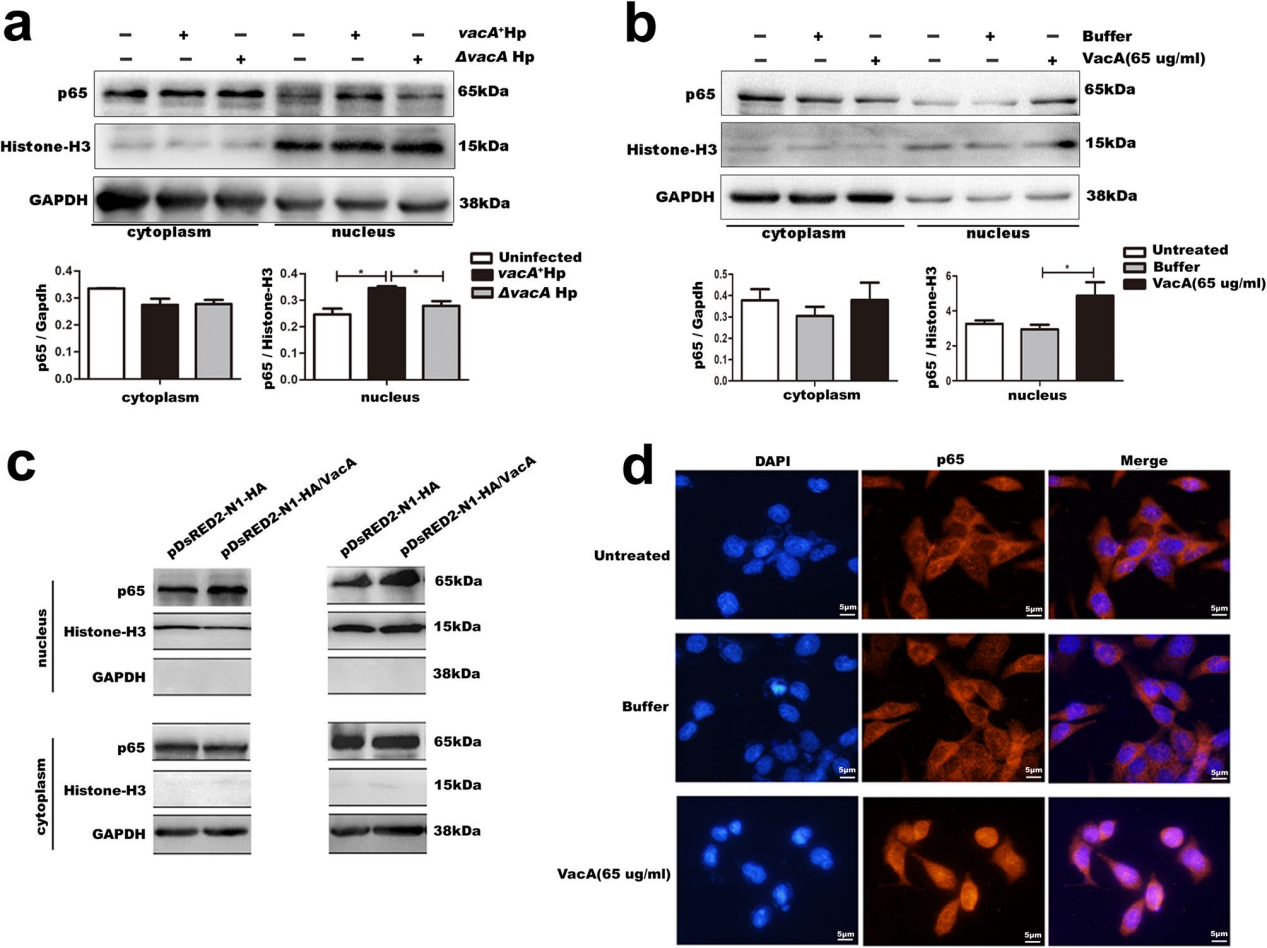
VacA activates the NF-κB pathway in GES-1 cells. **a-c** Cell fractionation and Western blot analyses were performed to assess the nuclear translocation of NF-κB P65. Extracted nucleocytoplasmic proteins were examined using histone H3 as a nuclear protein reference and GAPDH as a cytoplasmic protein reference and analysed with antibodies specific for p65. **d** Immunofluorescence microscopy analysis of the nuclear translocation of p65 in GES-1 cells. Cells were incubated with p65 antibody and fluorescent secondary antibodies, and nuclei were stained with DAPI. The images were obtained with a fluorescence inverted microscope. The red (representative of the area that contains p65) and blue (representative of the nucleus area that is conjugated to DAPI) images were overlaid to create a merged fluorescence in areas of colocalization (200 x magnification). **a** GES-1 cells were infected with *vacA*^*+*^Hp strain and Δ*vacA* Hp strain for 24 h, MOI = 20. The relative protein expression level of p65 (p65/Histone) in nucleus: *vacA*^*+*^Hp strain-infected group vs. Δ*vacA* Hp strain-infected group, *p* = 0.046. **b and d** GES-1 cells were incubated with VacA recombinant protein (65 µg/mL) for 12 h, and GES-1 cells incubated with isovolumetric protein buffer and cell culture medium served as a buffer control group and an untreated normal group, respectively. **b **The relative protein expression level of p65 (p65/Histone) in nucleus: recombinant VacA protein-incubated group vs. buffer-incubated group, *p* = 0.039. **c** GES-1 cells were transfected with pDsRED2-N1-HA/VacA for 48 or 72 h. Note: Comparisons were made between two groups using standard two-tailed Student’s t-test. The figure presents the average of three independent experiments (*n* = 3). Data are presented as mean ± SEM. Error bars represent standard error of mean. *: *p* < 0.05

To further verify the activation of the NF-κB signalling pathway by the Hp virulence factor VacA, we assessed the effect of VacA on the NF-κB signalling pathway in GES-1 cells via a subcellular fractionation assay, immunofluorescence analysis, and Western blotting. Nuclear–cytoplasmic fractionation followed by immunoblot analysis revealed a substantial increase in the nuclear p65 level in GES-1 cells after infection with the *vacA*^*+*^ Hp strain or treatment with recombinant VacA protein compared with that in the corresponding control group (Fig. 3a-b). Similar observations were made in GES-1 cells transfected with pDsRED2-N1-HA-VacA in parallel with pDsRED2-N1-HA for 48–72 h (Fig. 3c). Consistent with the Western blot results, immunofluorescence analysis revealed a significant increase in the nuclear entry of NF-κB p65 in GES-1 cells after recombinant VacA protein stimulation (Fig. 3d). Therefore, these results indicate that the Hp virulence factor VacA activates the NF-κB pathway in gastric epithelial cells.

### VacA induces apoptosis and inhibits proliferation of gastric epithelial cells

**Fig. 4:**
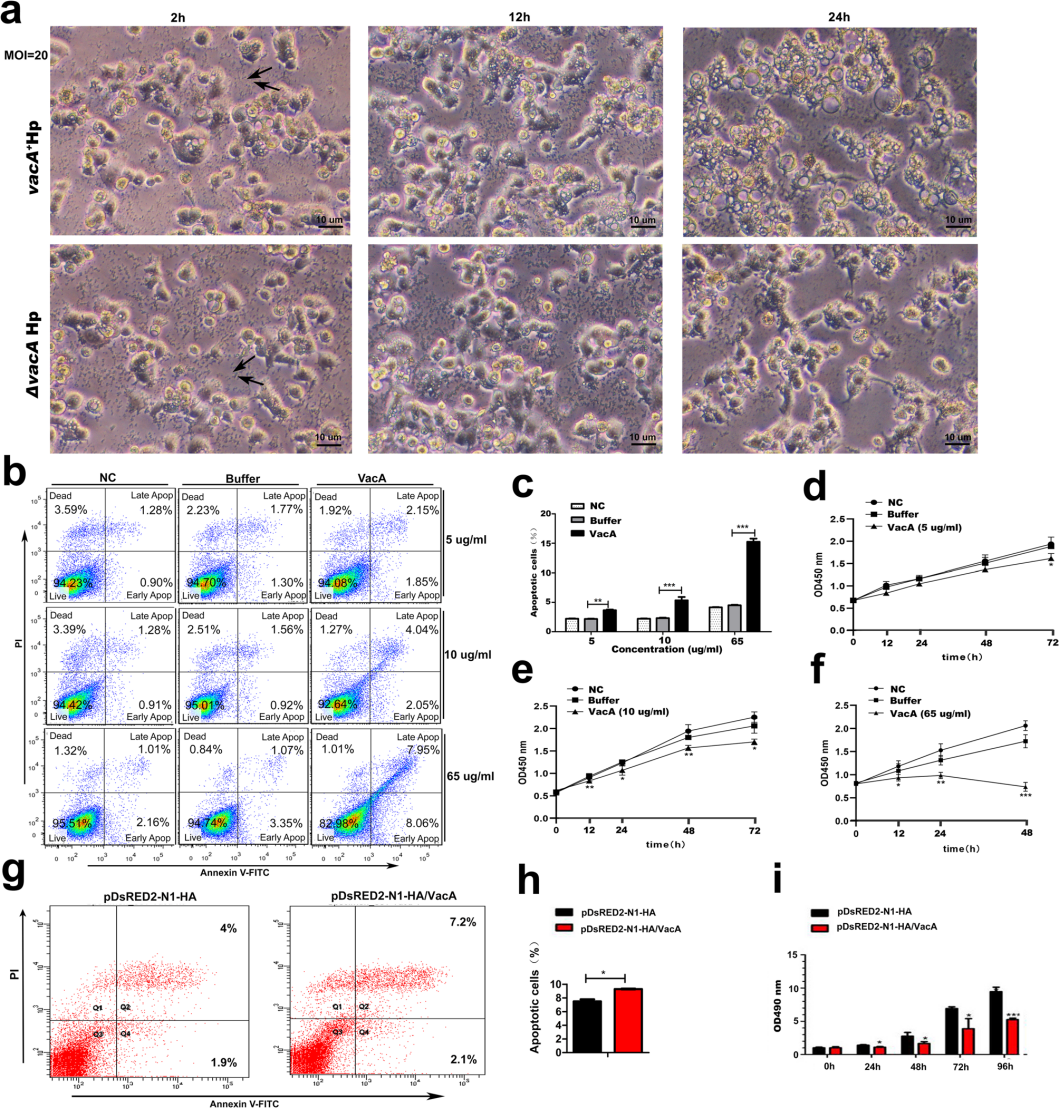
VacA induces apoptosis and inhibits proliferation of GES-1 cells. **a** The morphological changes in GES-1 cells after infection with the *vacA*^*+*^Hp strain or the Δ*vacA* Hp strain (MOI = 20) for 24 h were observed by inverted microscopy. Cellular morphological changes were visualized 2 h, 12 h and 24 h after infection by an inverted microscope (magnification × 100). Arrows indicate *Helicobacter pylori. ***b, c** GES-1 cells were incubated with VacA recombinant protein at a low concentration (5 µg/ml, 10 µg/ml) for 48 h and at a high concentration (65 µg/mL) for 12 h. In addition, GES-1 cells incubated with isovolumetric protein buffer and cell culture medium served as a buffer control group and an untreated normal group, respectively. Cellular apoptosis was analysed by Annexin V-FITC staining and flow cytometry. (c) Percentage of apoptotic cells were compared between recombinant VacA protein-incubated groups (at concentrations of 5, 10, and 65 µg/mL) and the correspondind buffer-incubated groups, with statistically significant differences (*p* = 0.008, *p* < 0.001, *p* < 0.001, respectively). **d-f** GES-1 cells were incubated with VacA recombinant protein (5, 10, and 65 µg/mL) for various durations (12–72 h). Cell viability was assessed by CCK8 assay. The OD450nm levels were compared between recombinant VacA protein-incubated groups (at a concentration of 5 µg/mL) and the corresponding buffer-incubated groups, with statistically significant differences observed at 72 h (*p* = 0.045). Levels of OD450nm were compared between recombinant VacA protein-incubated groups (at a concentration of 10 µg/mL) and corresponding buffer-incubated groups at 12, 24, 48, and 72 h, statistically significant differences were observed (*p* = 0.008, *p* = 0.047, *p* = 0.008, and *p* = 0.045, respectively). Levels of OD450nm were compared between recombinant VacA protein-incubated groups (at a concentration of 65 µg/mL) and corresponding buffer-incubated groups at 12, 24, and 48 h, statistically significant differences were observed (*p* = 0.039, *p* = 0.005, and *p* < 0.001, respectively). **g-h** Cellular apoptosis was analysed by Annexin V-FITC staining and flow cytometry. GES-1 cells were transfected with pDsRED2-N1-HA-VacA in parallel with pDsRED2-N1-HA for 48 h. (h) Percentage of apoptotic cells: pDsRED2-N1-HA/VacA-transfected group vs. empty vector pDsRED2-N1-HA-transfected group, *p* = 0.047. **i** Cell proliferation was assessed by an MTT assay. GES-1 cells were transfected with pDsRED2-N1-HA-VacA in parallel with pDsRED2-N1-HA at the indicated time points. Levels of OD490nm were compared between pDsRED2-N1-HA/VacA-transfected groups and empty vector pDsRED2-N1-HA-transfected groups at 24, 48, 72 and 96 h, statistically significant differences were observed (*p* = 0.044, *p* = 0.043, *p* = 0.038, *p* < 0.001, respectively). FITC, fluorescein isothiocyanate; PI, propidium iodide. Note: Comparisons were made between two groups using independent sample t tests. The figure presents the average of three independent experiments (*n* = 3). Data are presented as mean ± SEM. Error bars represent standard error of mean. *: *p* < 0.05. **: *p* < 0.01. ***: *p* < 0.001

Our previous results revealed that the toxin VacA increased the expression of the proapoptotic protein Bax and decreased the expression of the antiapoptotic protein Bcl-xl in gastric mucosal epithelial cells. We next demonstrated the proapoptotic effect of the toxin VacA via in vitro experiments. Firstly, we investigated the morphological changes in GES-1 cells after infection with the *vacA*^*+*^Hp strain or the Δ*vacA* Hp strain at an MOI of 20. After coculture with Hp for 24 h, significant cellular morphological changes indicative of apoptosis, including cytoskeletal disorganization, cytoplasmic vacuolization, karyorrhexis and apoptotic body formation, were observed in the *vacA*^*+*^Hp strain infection group compared with the Δ*vacA* Hp strain infection group (Fig. 4a). Flow cytometric analysis after Annexin V-FITC/PI staining following incubation with different concentrations of recombinant VacA protein for different durations (5 or 10 µg/ml for 48 h or 65 µg/ml for 48 h) revealed increased apoptosis of GES-1 cells in the recombinant VacA protein-treated group compared with the buffer-treated group (Fig. 4b-c). The CCK-8 assay revealed a significant reduction in the viability of GES-1 cells incubated with recombinant VacA protein over time (12–72 h) compared with the viability of cells in the buffer control group (Fig. 4d-f). Similar effects were observed in GES-1 cells transfected with pDsRED2-N1-HA-VacA in combination with pDsRED2-N1-HA for different durations (Fig. 4g-i). The results of the present study demonstrated that VacA has a biological effect of induces the apoptosis and inhibits the proliferation of gastric epithelial cells.

### Overexpression of TRAF1 promoted VacA-induced 4-1BB expression, NF-κB pathways activation, apoptosis-related molecules levels, and downstream inflammatory factors secretion in gastric epithelial cells, whereas silencing of TRAF1 led to the opposite effects

**Fig. 5:**
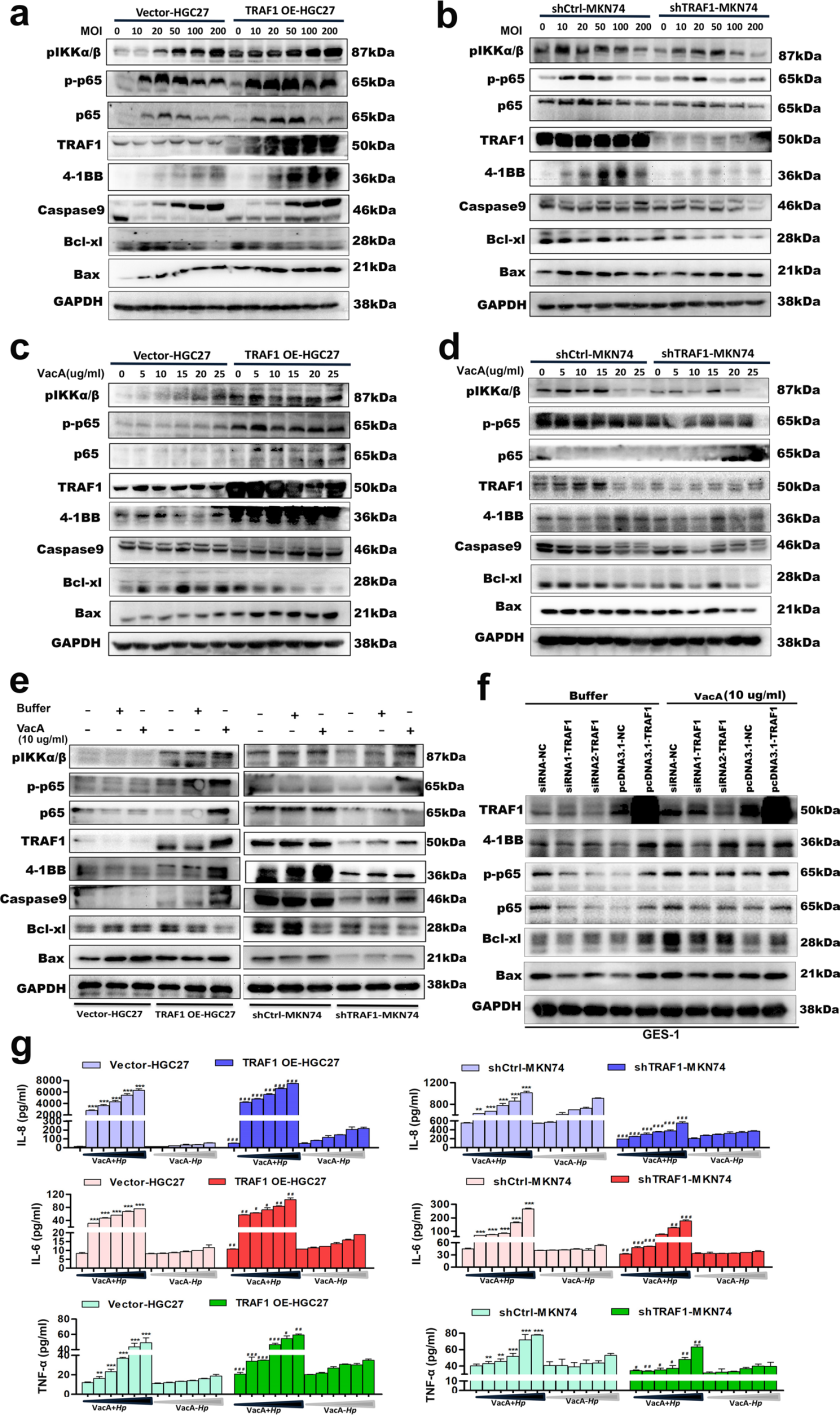
TRAF1 promotes the expression of 4-1BB, NF - κ B pathway proteins, apoptosis-related molecules and downstream inflammatory factors in gastric epithelial cells induced by VacA. **a, b** Western Blot was used to detect the protein expression levels of the target proteins (pIKKα/β, p-P65, p65, TRAF1, 4-1BB, caspase9, Bcl-xl and Bax) after co-culturing TRAF1 stably overexpressing/silenced cell lines with different concentrations of the *vacA*^+^Hp strain for 24 h. **c, d** Western Blot was used to detect the target proteins after co-incubation different concentrations of recombinant VacA protein with TRAF1 stably overexpressing/silenced cell lines for 48 h. **e** VacA protein (10ug/ml) was incubated with TRAF1 stably overexpressing/silenced gastric epithelial cells for 48 h, using an equal volume of acetic acid buffer as a control. Western blot was used to analyze the expression of various target molecules. **f** The transfected GES-1 cells were incubated with recombinant VacA protein (10 µg/ml) for 48 h. In addition, the transfected GES-1 cells incubated with isovolumetric protein buffer served as a buffer control group. Western blot was used to detect the effect of TRAF1 silencing or TRAF1 gene overexpression on the expression of the target proteins induced by recombinant VacA protein. **g** ELISA was used to analyze the secretion of IL-8, IL6, and TNF-αin the TRAF1 stably overexpressing/silenced cells supernatant after co-culturing with *vacA*^*+*^Hp strain or*ΔvacA* Hp strain at different concentrations for 24 h. Comparison of IL-8, IL-6, and TNF-αlevels between *vacA*^*+*^Hp strain-infected groups and *ΔvacA* Hp strain-infected groups in HGC27 and MKN74 cells under varying MOI conditions (MOI = 10,20,50,100,200) demonstrated statistically significant differences (*p* < 0.001 for all cytokine comparisons across both cell lines and all MOI gradients. *p < 0.05, **p < 0.01, ***p < 0.001.). When infected with *vacA*^*+*^Hp strain at different multiplicities of infection (MOIs: 10, 20, 50, 100, 200), comparisons of IL-8, IL-6, and TNF-αlevels between TRAF1-OE-HGC27 cells and Vector-HGC27 cells or between shTRAF1-MKN74 cells and shCtrl-MKN74 cells revealed statistically significant differences (all *p* < 0.05. #*p* < 0.05, ## *p* < 0.01, ### *p* < 0.001.). GAPDH was used as a protein internal reference. Vector-HGC27, TRAF1 OE-HGC27: HGC27 cells infected with control lentivirus or overexpressing lentivirus. shCtrl-MKN74, shTRAF1-MKN74: MKN74 cells infected with control lentivirus or interfering lentivirus. Note: Comparisons were made between two groups using standard two-tailed Student’s t-test. The figure presents the average of three independent experiments (*n* = 3). Data are presented as mean ± SEM. Error bars represent standard error of mean. shCtrl, short hairpin control; shTRAF1, short hairpin TRAF1; OE, overexpression

The aforementioned research findings suggest that VacA can enhance the expression of TRAF1, 4-1BB and NF-κB signaling pathway proteins, ultimately triggering apoptosis. Next, we used gene silencing or overexpression techniques to explore the role of TRAF1 in VacA induced abnormal proliferation and apoptosis of gastric epithelial cells. Firstly, we constructed a co-culture model using the *vacA*^+^Hp strain and stable TRAF1-overexpressing or TRAF1-silenced gastric epithelial cells. Western Blot analysis revealed that the overexpression of TRAF1 led to an upregulation of 4-1BB, NF-κB pathway proteins (p-P65 and pIKKα/β), and pro-apoptotic proteins (Caspase9 and Bax) in HGC27 cells infected with the *vacA*^+^Hp strain (Fig. 5a), and the above results showed strain concentration dependence. Conversely, silencing TRAF1 had the opposite effect, that is, silencing of TRAF1 attenuated the *vacA*^+^Hp strain-stimulated 4-1BB expression, NF-κB pathways activation, and pro-apoptotic proteins levels in MKN74 cells (Fig. 5b), and the above results showed strain concentration dependence. Moreover, we obtained the same results as co culturing strains in the subsequent co-incubation model using recombinant VacA protein and TRAF1 stable silenced or overexpressing human gastric epithelial cells (Fig. 5c-e).

To confirm the generality of our observations, we interfered with TARF1 expression in GES-1 cells using small interfering RNA (si-RNA) and obtained similar results through the same methodologies (Fig. 5f): After transfection with TRAF1-siRNA, the expression of TRAF1, 4-1BB, phospho-p65 and Bax was significantly downregulated in GES-1 cells. Incubation of TRAF1 gene-silenced cells with recombinant VacA protein rescued the levels of TRAF1, 4-1BB, phospho-p65 and Bax expression. Meanwhile, TRAF1 overexpression (TRAF1 OE) produced the opposite results in GES-1 cells. ELISA tests on the cell supernatant from the co-culture revealed a significant increase in the secretion of inflammatory factors IL-8, IL-6, and TNF-αin the *vacA*^+^Hp strain infection group compared to the Δ*vacA* Hp strain infection group, and this increase was concentration-dependent (Fig. 5g). TRAF1 overexpression enhances the secretion of inflammatory cytokines under the stimulation of Hp strain (Fig. 5g). Taken together, these results indicate that TRAF1 plays an important positive regulatory role in VacA-induced 4-1BB expression, NF-κB pathways activation, apoptosis-related molecules levels, and pro-inflammatory effects in gastric epithelial cells.

### Overexpression of TRAF1 enhanced VacA-induced NF-κB p65 nuclear translocation and cell apoptosis in gastric epithelial cells, whereas silencing of TRAF1 led to the opposite effects

**Fig. 6:**
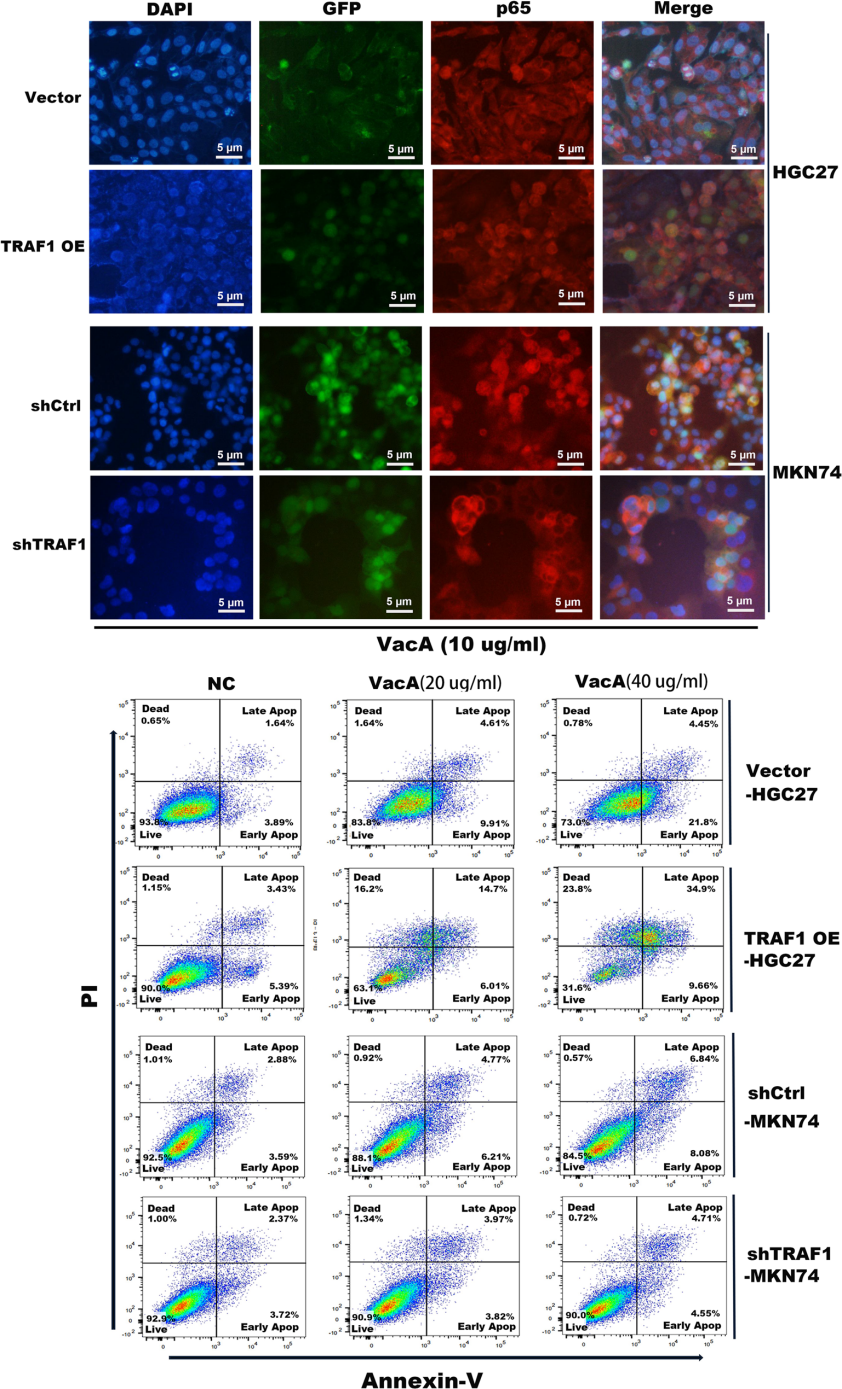
TRAF1 promotes the activation of the NF-κB pathway and cell apoptosis in gastric epithelial cells induced by VacA. **a **VacA recombinant protein (10 µg/mL) was incubated with TRAF1 stably overexpressing/silenced gastric epithelial cells for 48 h, and immunofluorescence was used to analyze NF-κB P65 nuclear translocation. DAPI (blue fluorescence) indicates the cell nucleus, GFP (green fluorescence) indicates the lentiviral fluorescent vector of the stable cell line, and P65 (red fluorescence). The image is magnified 200 times. **b** Different concentrations of VacA recombinant protein (20, 40 µg/mL) were incubated with TRAF1 stably overexpressing/silenced gastric epithelial cells for 48 h. Cells were stained with Annexin V/PI and apoptosis rates were detected by flow cytometry. Vector-HGC27, TRAF1 OE-HGC27: HGC27 cells infected with control lentivirus or overexpressing lentivirus. shCtrl-MKN74, shTRAF1-MKN74: MKN74 cells infected with control lentivirus or interfering lentivirus.shCtrl, short hairpin control; shTRAF1, short hairpin TRAF1; OE, overexpression

Previous data has shown that TRAF1 facilitates the levels of NF-κB pathways proteins and apoptosis-related molecules induced by VacA, we then employed cellular immunofluorescence and flow cytometry to analyze the effects of TRAF1 overexpression and silencing on NF-κB pathway activation and gastric epithelial cell apoptosis induced by VacA. As evident from our findings, the overexpression of TRAF1 enhances the transcriptional activation of NF-κB (manifested by increased P65 nuclear translocation) in HGC27 cells stimulated by recombinant VacA protein. Conversely, silencing TRAF1 could reduce NF-κB P65 nuclear translocation in MKN74 cells triggered by recombinant VacA protein (Fig. 6a & Supplementary Fig. 1). Additionally, flow cytometry analysis demonstrated that TRAF1 overexpression amplifies the apoptosis-inducing effects of recombinant VacA protein on HGC27 cells, leading to a notable surge in apoptotic cells. On the other hand, suppressing TRAF1 expression in MKN74 cells can diminish this apoptosis-inducing impact of recombinant VacA protein (Fig. 6b). These results suggest that, functionally, TRAF1 increased VacA-induced p65 nuclear translocation and transcriptional activity, leading to apoptosis.

### Suppression of the NF-κB signalling pathway counteracts the effect of VacA on the expression of TRAF1/4-1BB/IL-8 and apoptosis-related proteins and reduces the proapoptotic effect of VacA

**Fig. 7:**
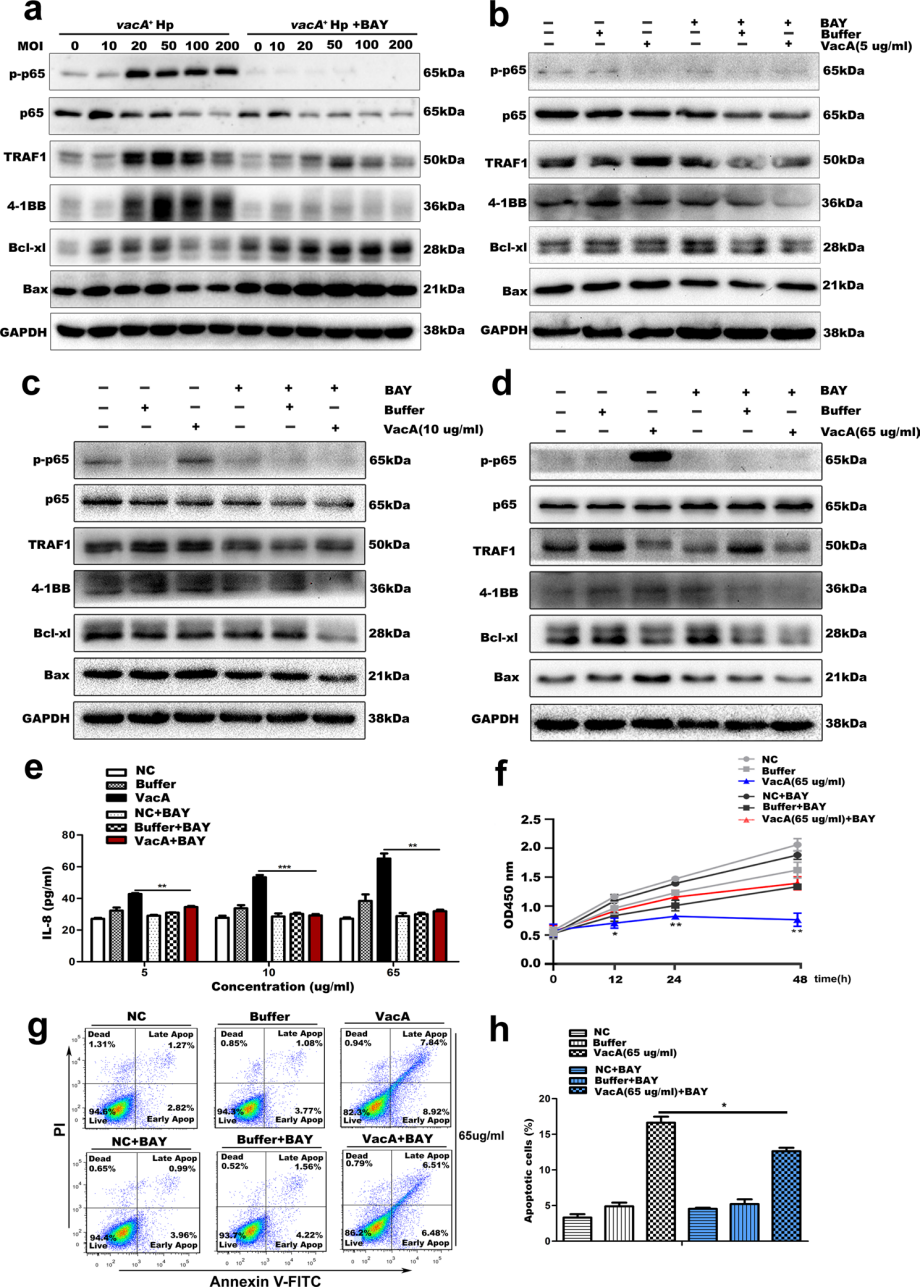
Suppression of the NF-κB signalling pathway counteracts changes in the expression of TRAF1/4-1BB/IL-8 and apoptosis-related proteins induced by VacA in GES-1 cells, resulting in a lower apoptosis rate and increased proliferation. GES-1 cells pretreated with BAY11-7082 (5 µM) were then infected with the *vacA*^*+*^ Hp strain at different MOIs (0, 10, 20, 50, 100, and 200) for 24 h or incubated with VacA recombinant protein at a low concentration (5, 10 µg/ml for 48 h) or at a high concentration (65 µg/ml for 12 h). Compared with the VacA recombinant protein incubation group, GES-1 cells incubated with isovolumetric protein buffer and cell culture medium served as a buffer control group and an untreated normal group, respectively. **a-d** The protein levels of the target molecules (TRAF1, 4-1BB, p65, phospho-p65, Bcl-xl and Bax) were determined by Western blot analysis. GAPDH was used as a loading control. **e** The secretion level of IL-8 in GES-1 cells after incubation with recombinant VacA protein was measured by ELISA. Comparison of IL-8 levels between recombinant VacA protein-incubated groups (at concentrations of 5, 10, and 65 µg/mL) and recombinant VacA protein + BAY11-7082-incubated groups revealed statistically significant differences at all three concentrations (*p* = 0.0045, *p* < 0.001, and *p* = 0.0034, respectively). **f** CCK-8 assay was used to assess the viability of GES-1 cells pretreated with BAY11-7082 and treated with recombinant VacA protein. OD450 levels were compared between groups incubated with 65 µg/mL recombinant VacA protein alone and those incubated with 65 µg/mL recombinant VacA protein + BAY11-7082 at 12, 24, and 48 h, statistically significant differences were observed at all time points (*p* = 0.041, *p* = 0.009, and *p* = 0.003, respectively). **g-h** Annexin V-FITC staining coupled with flow cytometry analysis of the apoptosis of GES-1 cells pretreated with BAY11-7082 and treated with VacA recombinant protein. (h) Percentage of apoptotic cells were compared between groups incubated with 65 µg/mL recombinant VacA protein alone and those incubated with 65 µg/mL recombinant VacA protein + BAY11-7082, with statistically significant differences (*p* = 0.037). FITC, fluorescein isothiocyanate; PI, propidium iodide. Note: Comparisons were made between two groups using standard two-tailed Student’s t-test. The figure presents the average of three independent experiments (*n* = 3). Data are presented as mean ± SEM. Error bars represent standard error of mean. *p < 0.05, **p < 0.01, ***p < 0.001. BAY, BAY11-7082

Based on the above-mentioned results, we speculated that the VacA–TRAF1–NF-κB pathway–IL-8 axis is involved in Hp induced abnormal proliferation and apoptosis of gastric epithelial cells. To explore whether the VacA-induced changes in the expression of TRAF1, 4-1BB, the chemokine IL-8 and apoptosis-related proteins are dependent on activation of the NF-κB pathway, we constructed a cell model of BAY11-7082-mediated NF-κB inhibition. Firstly, blocking NF-κB signalling with BAY11-7082 significantly attenuated the effect of the *vacA*^*+*^Hp trains infection on the expression of TRAF1, 4-1BB, and apoptosis-related proteins (Bcl-xl and Bax) in GES-1 cells (Fig. 7a), suggesting that the NF-κB pathway plays an important role in the pathogenesis of *vacA*^*+*^Hp infection. Moreover, we obtained same results as co culturing strains in the subsequent co-incubation model using different concentrations of recombinant VacA protein and GES-1 cells, and the results showed that treatment with BAY11-7082 decreased the level of phospho-p65 and that blockade of NF-κB signalling inhibited the changes in the expression of TRAF1, 4-1BB, and apoptosis-related proteins (Bcl-xl and Bax) and the secretion of IL-8 induced by the recombinant VacA protein in GES-1 cells (Fig. 7b-e). The CCK-8 assay and flow cytometry assay results demonstrated that treatment with BAY11-7082 significantly increased the proliferation and decreased the apoptosis rate of GES-1 cells compared with those in cells with recombinant VacA protein stimulation alone (Fig. 7f-h). These results suggest that the induction of TRAF1/4-1BB/IL-8 and apoptosis-related protein expression by VacA in gastric epithelial cells depends on NF-κB.

### BAY11-7082 or 4-1BB blocking antibody attenuated the promotive effect of TRAF1 overexpression on VacA-induced NF-κB pathway activation, apoptosis-related protein expression, and downstream inflammatory factors secretion in gastric epithelial cells, whereas 4-1BB agonist antibody reversed the inhibitory effect of TRAF1 silencing on VacA function

**Fig. 8:**
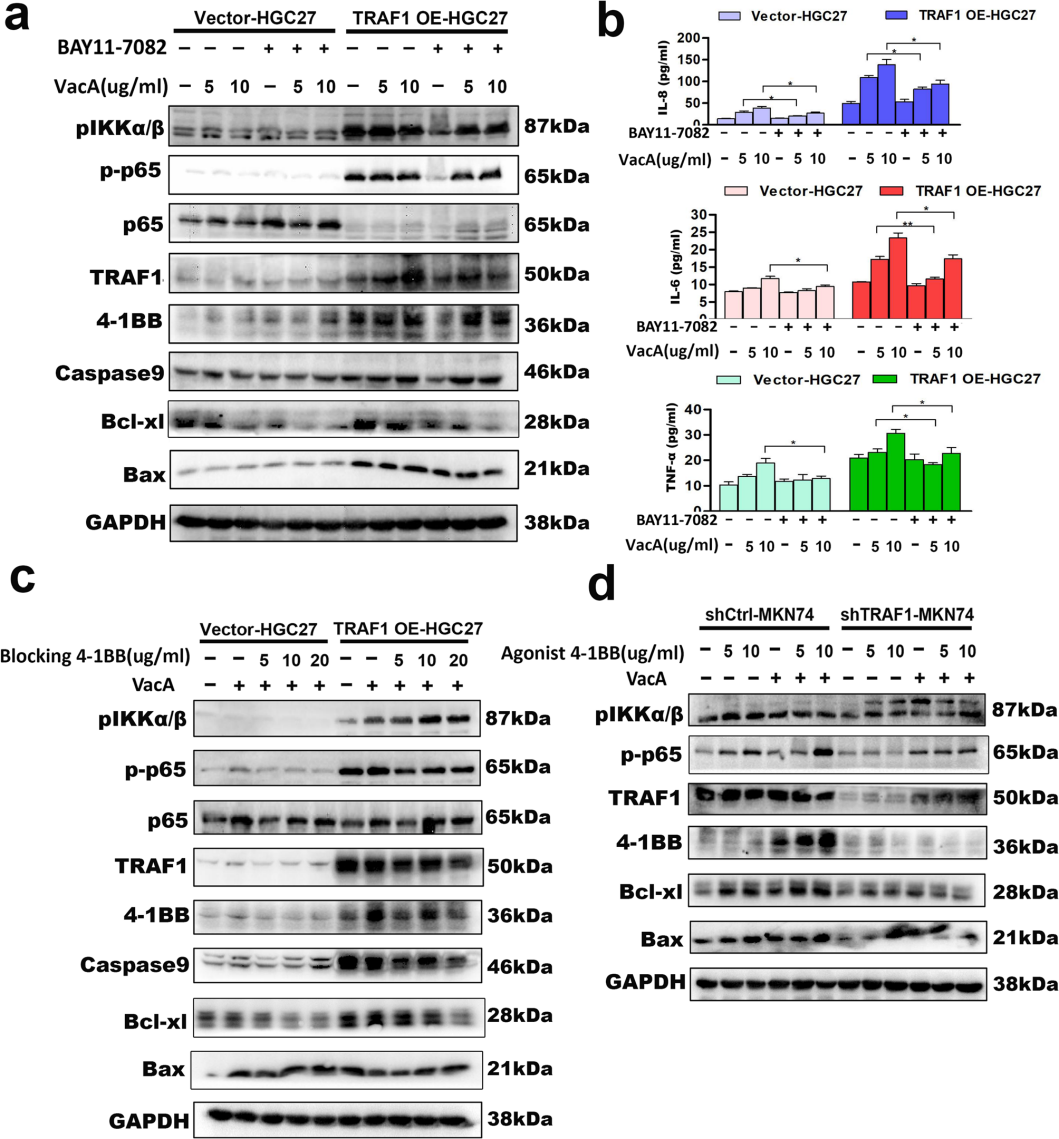
Effects of BAY11-7082 or 4-1BB blocking/agonist antibodies on TRAF1-mediated 4-1BB, NF-κB pathway activation, and apoptosis-related protein expression in the context of VacA action. **a, b** HGC27 cells (infected with TRAF1-overexpressing lentivirus or control lentivirus) pretreated with BAY11-7082 (5 µM) were incubated with VacA recombinant protein at different concentrations (5, 10 µg/ml) for 48 h. **a **Western blot analysis of target molecules (TRAF1, 4-1BB, pIKKα/β, p-P65, and apoptosis-related molecules Caspase9, Bcl-xl, Bax). **b** ELISA analysis of the secretion of inflammatory factors IL-8, IL6, and TNF-α in the cell supernatant. In Vector-HGC27 cells and TRAF1 OE-HGC27 cells, the levels of IL-8 were compared between groups treated with recombinant VacA protein (5 µg/mL and 10 µg/mL) and groups co-incubated with recombinant VacA protein and BAY11-7082; statistically significant differences were observed at both concentrations in Vector-HGC27 cells (*p* = 0.046 and *p* = 0.039, respectively) and in TRAF1 OE-HGC27 cells (*p* = 0.035 and *p* = 0.024, respectively).In Vector-HGC27 cells, the levels of IL-6 and TNF-α were compared between groups treated with recombinant VacA protein (5 µg/mL and 10 µg/mLL) and groups co-incubated with recombinant VacA protein and BAY11-7082; statistically significant differences were observed for both cytokines at the 10 µg/mL VacA protein concentration (IL-6: *p* = 0.040; TNF-α: *p* = 0.031). In TRAF1 OE-HGC27 cells, the levels of IL-6 and TNF-α were compared between groups treated with recombinant VacA protein (5 µg/mL and 10 µg/mL) and groups co-incubated with recombinant VacA protein and BAY11-7082; statistically significant differences were observed for both cytokines at both concentrations (IL-6: *p* = 0.008 and *p* = 0.041; TNF-α: *p* = 0.047 and *p* = 0.033). **c** HGC27 cells (infected with overexpressing lentivirus or control lentivirus) pretreated with 4-1BB blocking antibody at different concentrations (5, 10, 20ug/ml) were incubated with VacA recombinant protein (10 µg/ml) for 48 h, Western blot was used to analyze the expression of target molecules in the cells. **d** MKN74 cells (infected with TRAF1-silencing lentivirus or control lentivirus) pretreated with 4-1BB agonistic antibody at different concentrations (5, 10ug/ml) were incubated with VacA recombinant protein (10 µg/ml) for 48 h, Western blot was used to analyze the expression of target molecules in the cells. Note: Comparisons were made between two groups using standard two-tailed Student’s t-test. The figure presents the average of three independent experiments (*n* = 3). Data are presented as mean ± SEM. Error bars represent standard error of mean. *p < 0.05, **p < 0.01. shCtrl, short hairpin control; shTRAF1, short hairpin TRAF1; OE, overexpression; BAY, BAY11-7082

We then utilized gastric epithelial cell lines that stably overexpressed or silenced TRAF1, in conjunction with BAY11-7082 or 4-1BB blocking/inhibiting antibodies, to delve deeper into the effects of TRAF1 and 4-1BB on the NF-κB pathway activation induced by VacA. Western Blot combined with ELISA analysis indicated that suppressing the NF-κB pathway by BAY11-7082 could diminish the stimulatory effects of TRAF1 overexpression on 4-1BB, apoptosis-related proteins, and downstream inflammatory factors induced by VacA recombinant protein (Fig. 8a-b). As expected, the use of 4-1BB blocking antibodies can yield results similar to those observed in the NF-κB pathway inhibition model facilitated by BAY11-1082. Figure 8c demonstrated that the use of 4-1BB blocking antibodies attenuated the promotive effect of TRAF1 overexpression on VacA-induced NF-κB pathway activation, apoptosis-related protein expression in HGC27 cells. Conversely, the introduction of 4-1BB agonistic antibodies activates the NF-κB pathway in MKN74 cells, reversed the inhibitory effect of TRAF1 silencing on apoptosis-related proteins stimulated by VacA recombinant protein (Fig. 8d). Collectively, these results implicate the existence of a VacA-TRAF1-4-1BB-IL8 signaling axis in the abnormal proliferation-apoptosis observed in gastric epithelial cells infected with *Helicobacter pylori.*

### VacA promotes the expression of TRAF1, 4-1BB, and NF-κB p65 in the gastric mucosa of mice and promotes inflammatory damage to the gastric mucosa

**Fig. 9:**
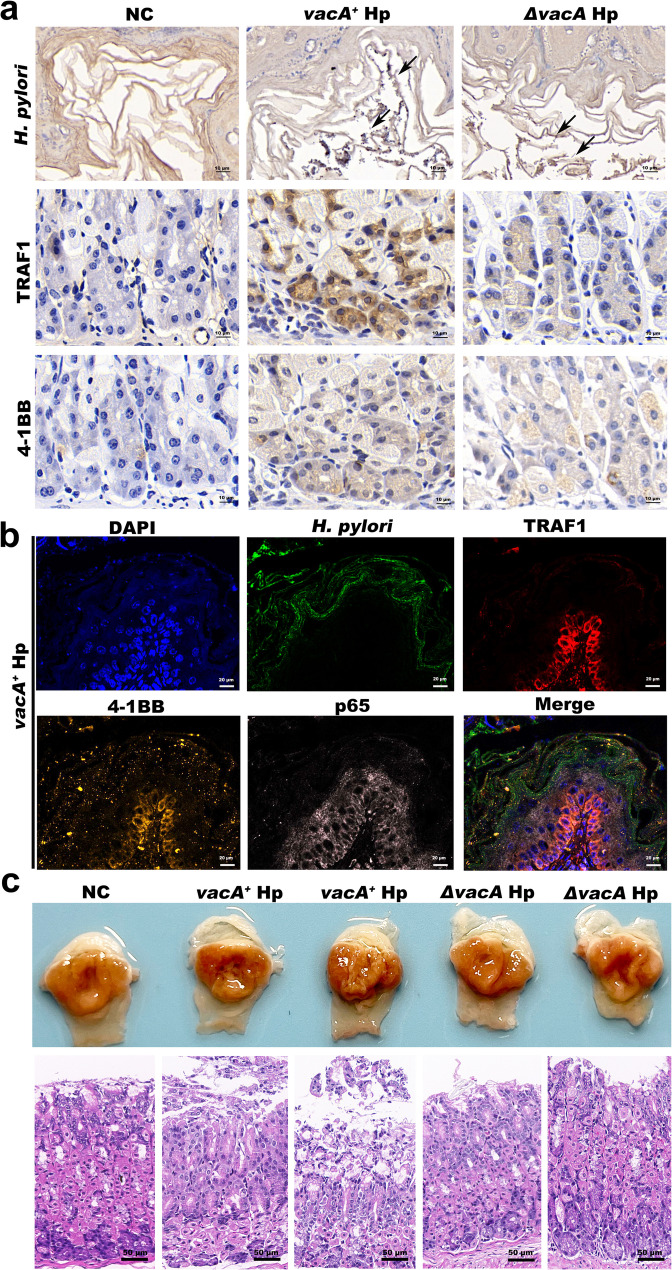
Constructing models of C57BL/6 mice infected with Hp. **a** Representative images of Hp, TRAF1 and 4-1BB immunohistochemical staining in gastric mucosal tissue from C57BL/6 mice infected with the *vacA*^*+*^Hp or*ΔvacA* Hp strains for one month. The black arrows indicate colonized Hp (80×). **b** Representative immunofluorescence images of the localization of Hp, TRAF1, 4-1BB, and P65 in gastric mucosal tissue of C57BL/6 mice infected with *vacA*^*+*^Hp strains for one month. DAPI (nuclei): blue; Hp: green; TRAF1: red; 4-1BB: orange; P65: pink. Magnification: 40×. **c** Gastric specimens and HE staining of gastric mucosal tissue from C57BL/6 mice infected with the *vacA*^*+*^Hp and*ΔvacA* Hp strains for twelve months. Magnification: 30×

Next, we investigated the impact of VacA on TRAF1, 4-1BB, and NF-κB p65 expression and inflammatory damage in mice gastric mucosal tissue, utilizing both wild-type and *vacA*-KO mutant Hp strains in vivo. C57BL/6 mice were infected with Hp for one month, we randomly selected three mice from each group to confirm the plantation of Hp in the gastric mucosa. As shown in the accompanying figure, both Hematoxylin and Eosin (HE), Giemsa and immunohistochemical staining confirmed the presence of numerous characteristic short, rod-shaped spiral Hp bacilli (indicated by black arrows) in the gastric mucosa of infected mice (Fig. 9a and Supplementary Fig. 1). Additionally, the rapid urease test indicated that in Hp-infected gastric mucosa, the urease reagent could be converted from a light yellow colour to a red colour, thus confirming the successful establishment of the C57BL/6 mice model of Hp infection (Supplementary Fig. 2). Consistent with the results in vitro, immunohistochemical staining display that there was a remarkable growth of TRAF1 and 4-1BB expression in the gastric mucosa of mice infected with *vacA*^*+*^Hp for one month compared to that in the uninfected control mice and mice infected with the*ΔvacA* Hp strain (Fig. 9a). Furthermore, we analysed the distribution of Hp, TRAF1, 4-1BB, and NF-κB P65 in gastric mucosal tissue using immunofluorescence staining. The accompanying figure demonstrates that Hp (indicated by green fluorescence) colonized the surface of gastric pits and that TRAF1, 4-1BB, and NF-κB P65 fluorescence was intensified at the site of Hp colonization (Fig. 9b). Twelve months after the C57BL/6 mice were gavaged with Hp strains, the gastric mucosal structure in mice infected with the *vacA*^*+*^Hp strain was disordered, with evident inflammatory cell infiltration, in contrast to the observations in uninfected control mice and mice infected with the*ΔvacA* Hp strain (Fig. 9c).

### Hp eradication or BAY11-7082 treatment could reverse VacA-induced expression of TRAF1 and inflammatory damage in mice gastric mucosal tissue

**Fig. 10:**
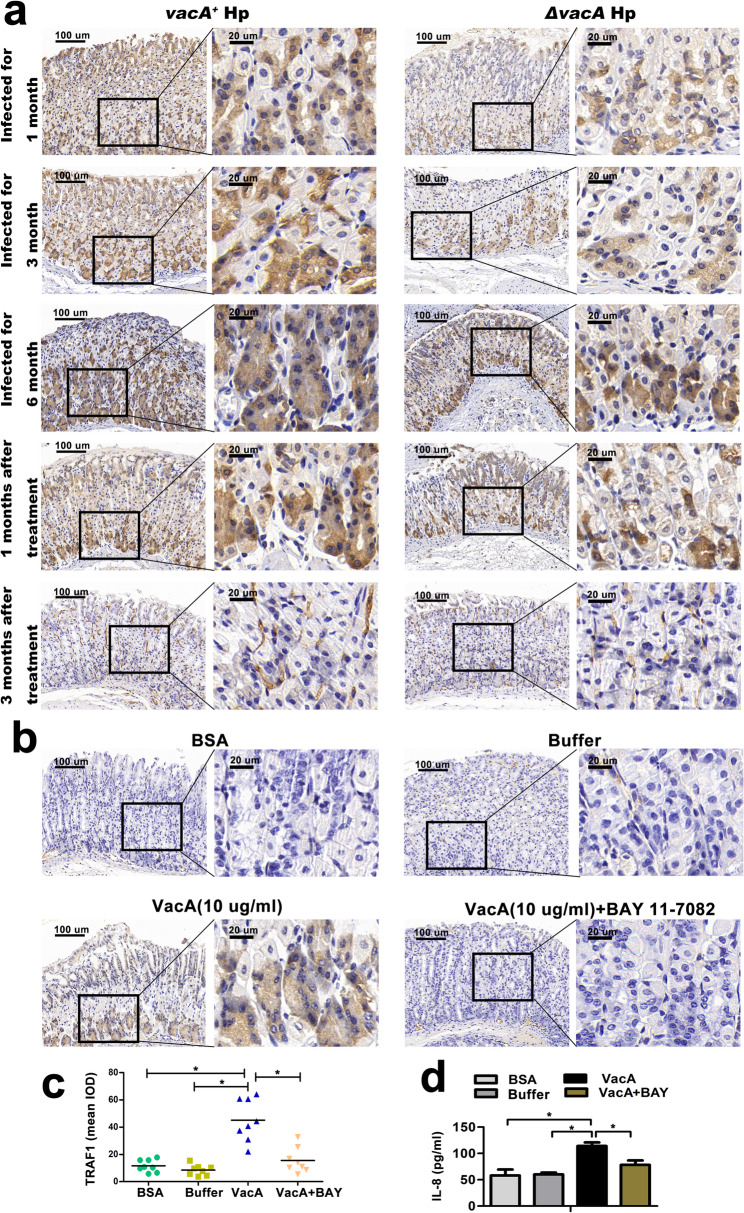
Expression of TRAF1 and IL-8 of C57BL/6 mice before and after Hp eradication or BAY11-7082 treatment. **a** C57BL/6 mice were treated with triple anti-Hp therapy six months after infection with the *vacA*^*+*^Hp or *ΔvacA* Hp strain. Immunohistochemical analysis of TRAF1 protein expression in gastric mucosal tissue from mice in the untreated, 1-month infected, 3-month infected, 6-month infected, 1-month post treatment, and 3-month post treatment groups. The figure shows representative images of immunohistochemical staining for TRAF1 in gastric mucosal tissue. Low magnification: 10×; high magnification: 40×. **b, d** Effect of BAY11-7082 treatment on TRAF1 expression in mouse gastric mucosal tissue and the serum IL-8 concentration from the mice gavaged with recombinant VacA protein. **b, c** Immunohistochemical analysis of TRAF1 expression in mouse gastric mucosal tissue from the BSA protein gavage group, protein hydrolysis solution (acetic acid) gavage group, recombinant VacA protein gavage group, and recombinant VacA protein gavage + BAY11-7082 intraperitoneal injection group; **c** ELISA was performed to quantify the serum IL-8 concentration in each group of mice. Low magnification: 10×; high magnification: 40×. **: *P* < 0.01. **c** The integrated optical density (IOD) values of TRAF1: recombinant VacA protein gavage group vs. BSA protein gavage group, *p* = 0.042; recombinant VacA protein gavage group vs. acetic acid-based buffer control group, *p* = 0.033; recombinant VacA protein gavage + BAY11-7082 intraperitoneal injection group vs. recombinant VacA protein gavage group, *p* = 0.046. **d** The levels of IL-8 of mouse: recombinant VacA protein gavage group vs. BSA protein gavage group, *p* = 0.039; recombinant VacA protein gavage group vs. acetic acid-based buffer control group, *p* = 0.035; recombinant VacA protein gavage + BAY11-7082 intraperitoneal injection group vs. recombinant VacA protein gavage group, *p* = 0.047. Note: Comparisons were made between two groups using standard two-tailed Student’s t-test. The figure presents the average of three independent experiments (*n* = 3). Data are presented as mean ± SEM. Error bars represent standard error of mean. *p < 0.05. BAY, BAY11-7082

C57BL/6 mice underwent triple anti-Hp therapy six months after infection with either the *vacA*^*+*^Hp or*ΔvacA* Hp strain. After 1 and 3 months of Hp eradication treatment, the efficacy of the anti-Hp therapy was verified through urease testing and Giemsa staining. Immunohistochemical examination indicated a notable decrease in TRAF1 expression post anti-Hp therapy, and the expression level of TRAF1 gradually normalized with extended treatment duration (Fig. 10a), correlating with a reduction in gastric mucosal inflammatory damage. Consistent with this finding, the expression levels of 4-1BB and Bcl-xl also exhibited progressive decreases after anti-Hp therapy compared to those in the untreated group (Supplementary Fig. 3). Additionally, the Western blot detection of TRAF1 and 4-1BB in mouse gastric mucosal tissue and ELISA analysis of IL-8 in mouse serum showed the same trend as immunohistochemistry (Supplementary Fig. 4).

Our in vitro studies showed that blocking the NF-κB pathway in gastric mucosal epithelial cells using BAY11-7082 decreases TRAF1 expression induced by VacA. Thus, we next sought to administer BAY11-7082 to mice and observe its impact on the effects of recombinant VacA protein in vivo. The results of our in vivo study mirrored those of our in vitro cell experiments. The immunohistochemical staining and ELISA revealed that the expression of TRAF1 and IL-8 of C57BL/6 mice treatment with recombinant VacA protein (10 mg/kg) for 14 days significantly increased compared with those mice treatment with BSA or buffer. Interestingly, intraperitoneal injection of BAY11-7082 reversed the increase of TRAF1 induced by recombinant VacA protein in mice (Fig. 10b), which is consistent with in vitro experiments.

## Discussion

The results of the present study revealed that infection with a *vacA*-positive Hp strain, incubation with recombinant VacA protein and transfection of the pDsRED2-N1-HA/VacA plasmid changed the expression of TRAF1, upregulated the expression of 4-1BB and the inflammatory factor IL-8, and activated the NF-κB pathway by facilitating P65 nuclear transport, which promoted apoptosis and inhibited cell proliferation. Moreover, overexpression of TRAF1 promoted VacA-induced 4-1BB expression, NF-κB pathways activation, apoptosis-related molecules levels, and downstream inflammatory factors secretion in gastric epithelial cells, whereas silencing of TRAF1 led to the opposite effects. BAY11-7082 or 4-1BB blocking antibody attenuated the promotive effect of TRAF1 overexpression on VacA-induced NF-κB pathway activation, apoptosis-related protein expression, and downstream inflammatory factors secretion in gastric epithelial cells, whereas 4-1BB agonist antibody reversed the inhibitory effect of TRAF1 silencing on VacA function. In vivo, the immunofluorescence of TRAF1, 4-1BB and NF-κB p65 at *vacA*^+^Hp colonization sites was increased, and TRAF1 expression increased gradually with prolonged infection, correlating with exacerbated gastric mucosal inflammation and damage. Hp eradication or BAY11-7082 treatment reversed TRAF1 expression in mouse mucosal tissue. Our data indicated that the VacA–TRAF1–NF-κB pathway–IL-8 axis may be involved in the abnormal proliferation and apoptosis of gastric epithelial cells induced by Hp infection. TRAF1 and 4-1BB might thus be promising therapeutic targets for Hp-mediated gastric “inflammatory carcinoma”, and our findings provide insights into the mechanism through which Hp causes inflammation and cancer.

VacA is secreted into the extracellular space by Hp through the type V secretion system (T5SS), after which it binds to receptors on the surface of host cells and is internalized to elicit several different effects on host cells, including cellular vacuolation, membrane potential depolarization, mitochondrial dysfunction, autophagy, mitogen-activated protein kinase activation and the inhibition of T-cell activation and proliferation (Ansari and Yamaoka 2020; Palframan et al. 2012; Chauhan et al. 2019; Foegeding et al. 2016). In addition, VacA can induce the death of gastric epithelial cells. Thus far, many studies have suggested that VacA induces gastric epithelial cell apoptosis. Cho SJ et al. reported that recombinant VacA inhibits carcinoma cell growth in a dose- and time-dependent manner, is a potent inducer of apoptosis, and mediates the development of gastric diseases through G1 arrest (Cho et al. 2003). Radin et al. proposed a “commander” role for VacA in Hp-induced apoptosis and reported that AZ-521 cell death induced by VacA resulted in Poly (ADP-ribose) polymerase (PARP) activation and the release of Lactate Dehydrogenase (LDH) and High Mobility Group Box 1 (HMGB1, a proinflammatory protein) (Radin et al. 2011). Consistent with these findings, our results provide further evidence that VacA promotes the apoptosis and inhibits the proliferation of gastric epithelial cells.

Current studies suggest that VacA-induced apoptosis may play a key role in gastric carcinogenesis by increasing cell proliferation and/or causing gastric atrophy. Increased apoptosis may serve as a stimulus for a compensatory hyperproliferative and potentially preneoplastic response in the context of chronic Hp infection (Moss et al. 1996; Anti et al. 1998). The mechanism responsible for VacA-induced apoptosis has always been considerably controversial. Many studies have shown that VacA can be translocated to mitochondria, where it causes dissipation of the DWm, cytochrome c release, and activation of the proapoptotic factor Bax, thereby leading to apoptosis, (Jain et al. 2011; Galmiche et al. 2000; Yamasaki et al. 2006) but the mechanism through which VacA induces Bax activation is not fully understood. Another study showed that VacA reduces the expression of prosurvival factors (Matsumoto et al. 2011) and causes endoplasmic reticulum (ER) stress (Akazawa et al. 2013), which could also contribute to VacA-induced cell death. Our data showed that VacA promotes apoptosis and inhibits the proliferation of gastric epithelial cells via activation of the TRAF1/4-1BB/NF-kappaB pathway.

TRAFs were initially discovered as adaptor proteins that bind to tumour necrosis factor receptor family members, which are thought to be important regulators of signalling pathways related to cell death and cellular responses to stress (Bradley and Pober 2001). TRAF1 is an NF-κB-inducible protein, and the absence of a RING domain in its N-terminal region, which is present in other TRAFs, makes TRAF1 unique among members of the TRAF family (Edilova et al. 2018). TRAF1 amplifies survival signalling downstream of certain TNFR family members, including 4-1BB, by promoting canonical NF-κB and Mitogen-Activated Protein Kinase (MAPK) activation (McPherson et al. 2012). Studies have shown that TRAF1 can inhibit apoptosis induced by activation of the TNF receptor or the T-cell receptor. In addition, TRAF1 is involved in regulating the activation of NF-κB and exerts an antiapoptotic effect on lymphoma cells (Sabbagh et al. 2006; Guo et al. 2009). However, in contrast with these observations, TRAF1 has also been reported to be a target of caspases, which become activated during apoptosis induced by TNF family death receptors (Leo et al. 2001). TRAF1 can also initiate apoptosis by recruiting Fas-associated death domain proteins (Chaudhary et al. 2000; Gupta 2003) and promote apoptosis and inflammatory responses in multisystem diseases (Zhang et al. 2014; Xu et al. 2019; Rajandram et al. 2012; Lu et al. 2013). However, information on the role of TRAF1 in the regulation of gastric “inflammatory carcinoma” lesions mediated by the Hp virulence factor VacA is limited. We found that both the exogenous stimulation of gastric epithelial cells with a *vacA*-positive Hp strain or recombinant VacA protein and the endogenous expression of VacA in gastric epithelial cells upregulated the expression of TRAF1, 4-1BB and the inflammatory factor IL-8; activated the NF-κB pathway; and promoted apoptosis. Recombinant VacA protein-induced TRAF1 expression and the ability of TRAF1 to promote apoptosis and inhibit proliferation were found to be regulated by NF-κB activation. Hp infection-initiated gastric inflammation is believed to play an important role in Hp-induced carcinogenesis. The virulence factors of Hp interact with host receptors or targets, leading to the activation of inflammatory signalling pathways (e.g., the NF-κB signalling pathway) and the subsequent release of proinflammatory cytokines, which is a major event in the initiation and development of gastric cancer. (Wang et al. 2014; Lamb et al. 2013; Kalali et al. 2014). Previous studies have shown that VacA can promote the release of the inflammatory factor IL-8 by activating the NF-κB pathway (Kalali et al. 2014; Takeshima et al. 2009), consistent with some of our research results. Wan et al. reported that the expression of TRAF1 in response to Hp infection was strongly related to the Hp virulence factor CagA and that the upregulation of TRAF1 exerts antiapoptotic effects on Hp-infected gastric cells (Wan et al. 2016). In fact, reciprocal regulation of VacA- and CagA-mediated signalling has been reported (Tegtmeyer et al. 2009; Argent et al. 2008). However, the underlying mechanism needs to be further clarified.

Notably, the expression of TRAF1 was found to be decreased under infection with a *vacA*-positive Hp strain at a high concentration or for an extended duration, and a similar result was found after incubation with the recombinant VacA protein; that is, the expression of TRAF1 was decreased following stimulation with a high concentration of recombinant VacA protein. It has been speculated that incubation with high concentrations of the toxin VacA activates the NF-κB signalling pathway in cells and that the bypass of apoptotic pathways, such as those driven by Caspase-8 or TNF-α, may occur simultaneously. VacA may participate in the cleavage of TRAF1 mediated by Cysteine Aspartic Acid Specific Protease 8 (Caspase-8) or TNF-α, (Chaudhary et al. 2000; Irmler et al. 2000; Jang, et al. 2001) resulting in a reduction in the TRAF1 level after its degradation and a subsequent increase in apoptosis through the regulation of the expression of proapoptotic Bcl-2 family members such as Bcl-xl and Bax. The NF-κB pathway has a dual regulatory effect on apoptosis, both inhibiting and promoting it. The direction of this regulation is related to multiple factors, such as the application of different stimuli, the length of the stimulation time, the cell type, and the number of NF-κB family members (Karin and Lin 2002). As observed in our present and previous studies, VacA induced activation of the NF-κB pathway and promoted the apoptosis and inhibited the proliferation of gastric epithelial cells. However, further studies are needed to clarify the intracellular signalling machinery that controls VacA-induced apoptosis.

Taken together, these findings suggest a working model for the important role of TRAF1 in the abnormal proliferation and apoptosis of gastric mucosal epithelial cells caused by the Hp virulence factor VacA. In this model, the Hp virulence factor VacA alters the expression of TRAF1, which then upregulates 4-1BB expression, leading to NF-κB signalling pathway activation. The proinflammatory cytokine IL-8 is subsequently released, which upregulates proapoptotic proteins such as Bax and downregulates antiapoptotic proteins such as Bcl-xl, and these effects ultimately promote apoptosis and inhibit cell proliferation. In conclusion, our data indicate that the VacA–TRAF1–NF-κB pathway–IL-8 axis may be involved in the abnormal proliferation and apoptosis of gastric epithelial cells induced by Hp infection (Supplementary Fig. 5). TRAF1 and 4-1BB might thus be promising therapeutic targets for Hp-mediated gastric “inflammatory carcinoma”, providing insights into the mechanism through which Hp causes inflammation and cancer.

## Supplementary Information

Supplementary Material 1.

## Data Availability

The raw data of the RNA-seq was deposited in the GEO database (GSE290844). The other data supporting the findings of this study are available from the corresponding author upon reasonable request.

## Abbreviations

4-1BB (CD137, TNFRSF9): tumour necrosis factor receptor superfamily 9
ATCC: American Type Culture Collection
Bax: Bcl-2-associated X protein
BAY: BAY11-7082
Bcl-2: Bcl-2 apoptosis regulator
Bcl-xl: Bcl-2-like 1
BSA: Bovine Serum Albumin
Caspase3: Cysteine Aspartic Acid Specific Protease 3
Caspase8: Cysteine Aspartic Acid Specific Protease 8
Caspase9: Cysteine Aspartic Acid Specific Protease 9
CCK-8: Cell Counting Kit-8
CFU: Colony Forming Unit
DAB: 3,3’-Diaminobenzidine
DAPI: 4,6-diamidino-2-phenylindole
EDTA: Ethylene Diamine Tetraacetic Acid
ELISA: Enzyme-Linked Immunosorbent Assay
ER: Endoplasmic reticulum
FITC: Fluorescein Isothiocyanate
GAPDH: Glyceraldehyde-3-phosphate dehydrogenase
GEO: Gene Expression Omnibus
GFP: Green fluorescence
GSEA: Gene Set Enrichment Analysis
HE: Hematoxylin and Eosin
Hp: *Helicobacter pylori*
HMGB1: High Mobility Group Box 1
IOD: Integrated Optical Density
IL-6: Interleukin 6
IL-8: Interleukin 8
KEGG: Kyoto Encyclopedia of Genes and Genomes
KO: Knockout
LDH: Lactate Dehydrogenase
MAPK: Mitogen-Activated Protein Kinase
MOI: multiplicities of infection
MTT: 3-(4,5-Dimethylthiazol-2-yl)-2,5-diphenyltetrazolium Bromide
NC: Negative control
NF-κB: Nuclear factor κB
NIK: NF-κB-inducing kinase
OD: Optical density
OE: Overexpression
PBS: Phosphate Buffered Saline
PCR: Polymerase Chain Reaction
PI: Propidium iodide
PARP: Poly(ADP-ribose) polymerase
qRT–PCR: Quantitative real-time polymerase chain reaction
RNA-Seq: RNA Sequencing
SDS–PAGE: Sodium Dodecyl Sulfate-Polyacrylamide Gel Electrophoresis
shCtrl: Short hairpin control
shRNA: Short Hairpin RNA
shTRAF1: Short hairpin TRAF1
si-RNA: Small interfering RNA
SPSS: Statistical Package for the Social Sciences
STR: Short tandem repeat
TNF-α: Tumour necrosis factor alpha-like
TNFR: Tumor Necrosis Factor Receptor
TRAF1: TNF receptor associated factor 1
VacA: Vacuolating cytotoxin A
*vacA*^+^Hp strain: Wild-type strain Hp American Type Culture Collection 26695
Δ*vacA* Hp strain: *vacA*-KO mutant Hp American Type Culture Collection 26695
WHO: World Health Organization
