# Supplementary material for: Helicobacter pylori VacA modulates TRAF1-mediated 4-1BB/NF-kappaB axis to induce host apoptosis and chronic inflammatory damage
Source: Mol Med. 2025 Oct 24;31:317. doi: 10.1186/s10020-025-01349-5 (PMC12551314; doi:10.1186/s10020-025-01349-5)
Supplement: Supplementary file 1 — Supplementary Material 1. [file 10020_2025_1349_MOESM1_ESM.docx]

**Supplementary Materials**


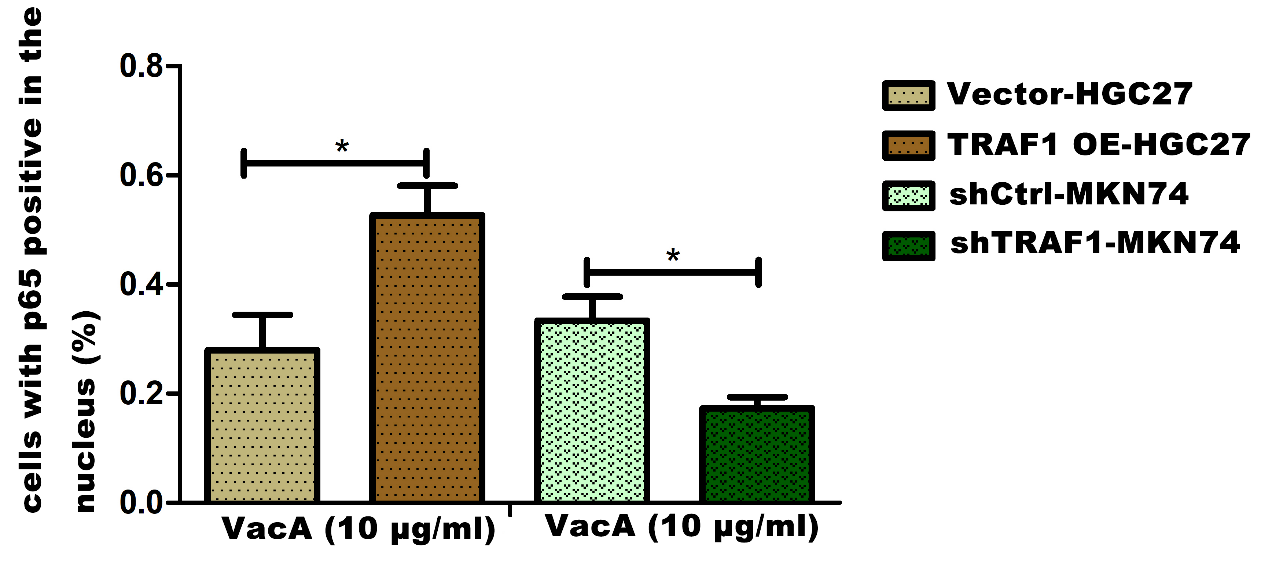


**Supplementary Fig 1.** TRAF1 promotes VacA-induced NF-κB p65 protein from cytoplasm to nucleus in gastric epithelial cells. VacA recombinant protein (10µg/ml) was incubated with TRAF1 stably overexpressing/silenced gastric epithelial cells for 48 hours, and immunofluorescence was used to analyze NF-κB P65 nuclear translocation. Quantitative analysis of the cells with NF-κB positive in the nucleus. Cells with p65 positive in the nucleus: vector-HGC27 vs. TRAF1 OE-HGC27, *p*=0.043; shCtrl-MKN74 vs. shTRAF1-MKN74, *p*=0.030. Vector-HGC27, TRAF1 OE-HGC27: HGC27 cells infected with control lentivirus or overexpressing lentivirus. shCtrl-MKN74, shTRAF1-MKN74: MKN74 cells infected with control lentivirus or interfering lentivirus. Note: Comparisons were made between two groups using standard two-tailed Student's t-test. The figure presents the average of three independent experiments (n=3). Data are presented as mean±SEM. Error bars represent standard error of mean. *: *p*<0.05. shCtrl, short hairpin control; shTRAF1, short hairpin TRAF1; OE, overexpression.


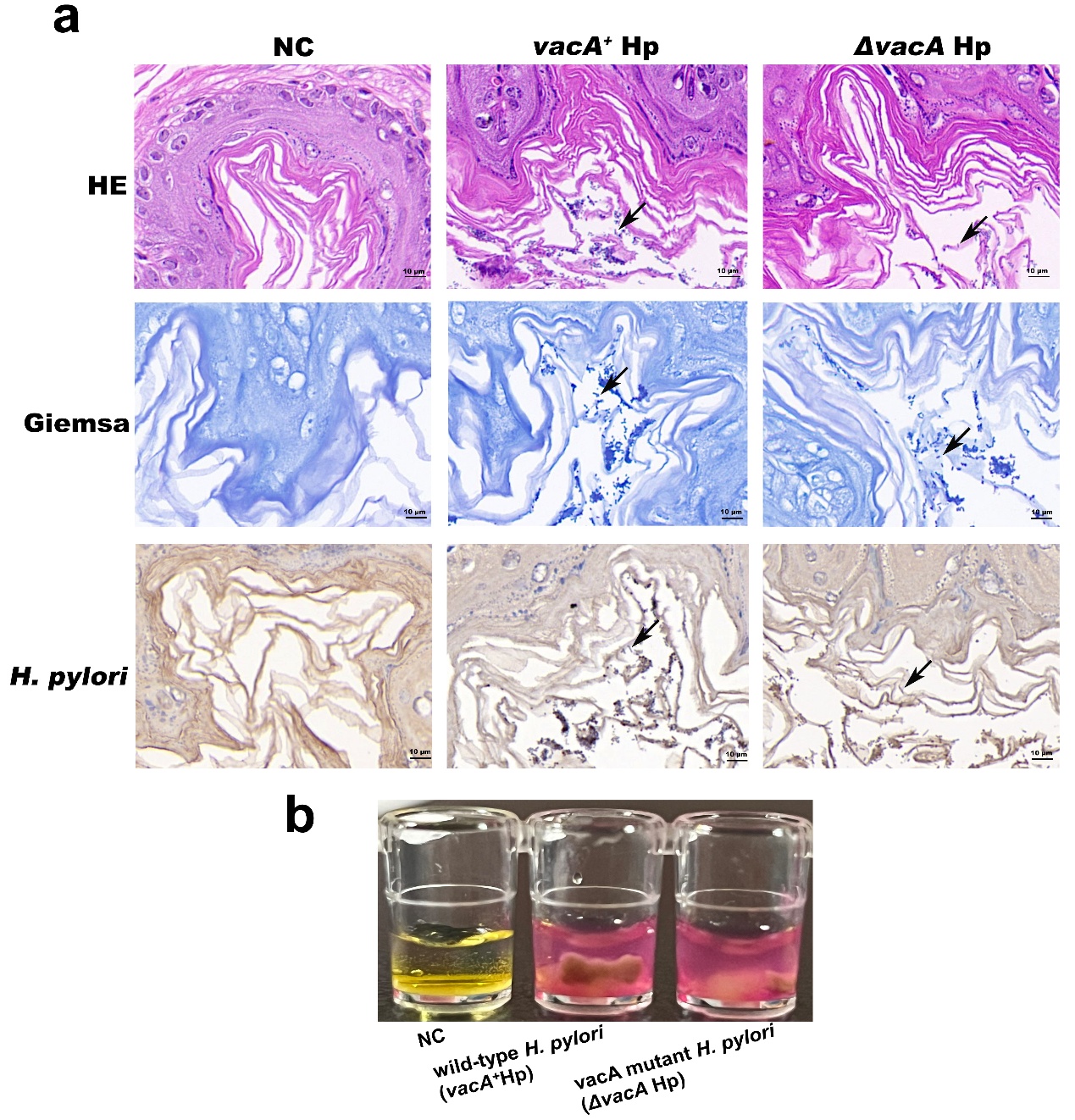


**Supplementary Fig 2.** Constructing models of C57BL/6 mice infected with Hp. **a.** Representative images of HE, Giemsa and immunohistochemical staining of Hp in gastric mucosal tissue from C57BL/6 mice infected with the *vacA^+^*Hp or*ΔvacA* Hp strains for one month. The black arrows indicate colonized Hp (80×). **b.** The rapid urease test of gastric mucosal tissue from mice.


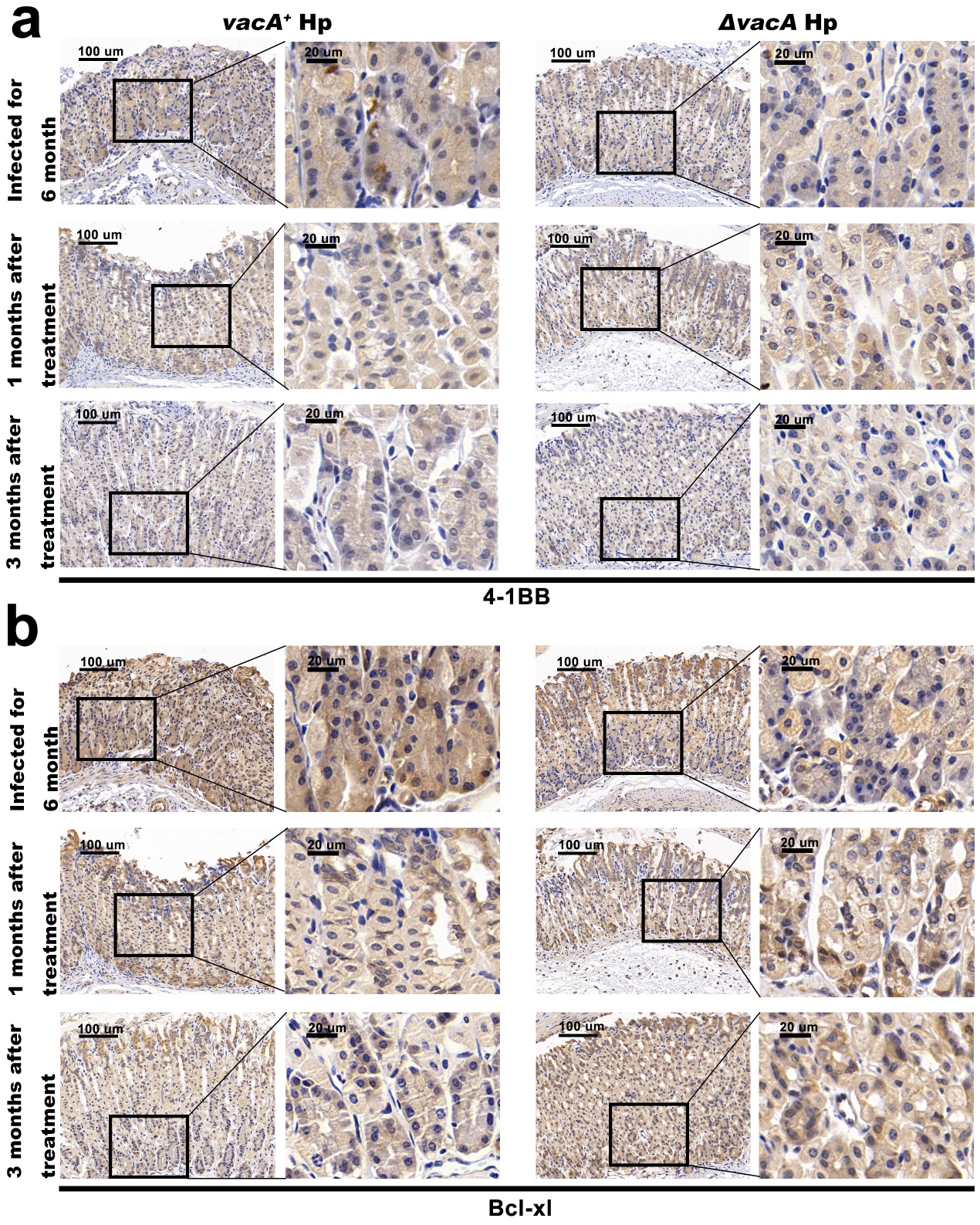


**Supplementary Fig 3.** Expression of 4-1BB and Bcl-xl of C57BL/6 mice before and after Hp eradication. C57BL/6 mice were treated with triple anti-Hp therapy six months after infection with the *vacA^+^*Hp or*ΔvacA* Hp strain. Immunohistochemical analysis of 4-1BB and Bcl-xl protein expression in gastric mucosal tissue from mice in the 6-month infected, 1-month post treatment, and 3-month post treatment groups. The figure shows representative images of immunohistochemical staining for 4-1BB and Bcl-xl in gastric mucosal tissue. Low magnification: 10×; high magnification: 40×.





**Supplementary Fig 4.** Expression of TRAF1, 4-1BB, Bcl-xl and IL-8 of C57BL/6 mice before and after Hp eradication. C57BL/6 mice were treated with triple anti-Hp therapy six months after infection with the *vacA^+^*Hp or*ΔvacA* Hp strain. **a-b.** Western blotting analysis of TRAF1, 4-1BB and Bcl-xl protein expression in gastric mucosal tissue from mice in the untreated and 3-month post treatment groups. **c.** ELISA analysis of the serum IL-8 concentration in each group of mice. The level of IL-8 of mouse: *vacA^+^*Hp strain-infected group vs. Blank control group, *p*<0.001; *vacA^+^*Hp strain-infected group vs. Δ*vacA* Hp strain-infected group, *p*=0.042; *vacA^+^*Hp strain-infected untreated group vs. *vacA^+^*Hp strain-infected treated group, *p*=0.007; Δ*vacA* Hp strain-infected untreated group vs. Δ*vacA* Hp strain-infected treated group, *p*=0.039. Note: Comparisons were made between two groups using standard two-tailed Student's t-test. The figure presents the average of three independent experiments (n=3). Data are presented as mean±SEM. Error bars represent standard error of mean. *: *p*<0.05. **; *p*<0.01. ***; *p*<0.001.
